# Analysis of Hierarchical Organization in Gene Expression Networks Reveals Underlying Principles of Collective Tumor Cell Dissemination and Metastatic Aggressiveness of Inflammatory Breast Cancer

**DOI:** 10.3389/fonc.2018.00244

**Published:** 2018-07-04

**Authors:** Shubham Tripathi, Mohit Kumar Jolly, Wendy A. Woodward, Herbert Levine, Michael W. Deem

**Affiliations:** ^1^PhD Program in Systems, Synthetic, and Physical Biology, Rice University, Houston, TX, United States; ^2^Center for Theoretical Biological Physics, Rice University, Houston, TX, United States; ^3^Department of Radiation Oncology, The University of Texas MD Anderson Cancer Center, Houston, TX, United States; ^4^MD Anderson Morgan Welch Inflammatory Breast Cancer Research Program and Clinic, The University of Texas MD Anderson Cancer Center, Houston, TX, United States; ^5^Department of Bioengineering, Rice University, Houston, TX, United States; ^6^Department of Physics and Astronomy, Rice University, Houston, TX, United States

**Keywords:** collective dissemination, inflammatory breast cancer, epithelial-to-mesenchymal transition, hierarchy, hybrid E/M, cophenetic correlation coefficient

## Abstract

Clusters of circulating tumor cells (CTCs), despite being rare, may account for more than 90% of metastases. Cells in these clusters do not undergo a complete epithelial-to-mesenchymal transition (EMT), but retain some epithelial traits as compared to individually disseminating tumor cells. Determinants of single cell dissemination versus collective dissemination remain elusive. Inflammatory breast cancer (IBC), a highly aggressive breast cancer subtype that chiefly metastasizes *via* CTC clusters, is a promising model for studying mechanisms of collective tumor cell dissemination. Previous studies, motivated by a theory that suggests physical systems with hierarchical organization tend to be more adaptable, have found that the expression of metastasis-associated genes is more hierarchically organized in cases of successful metastases. Here, we used the cophenetic correlation coefficient (CCC) to quantify the hierarchical organization in the expression of two distinct gene sets, collective dissemination-associated genes and IBC-associated genes, in cancer cell lines and in tumor samples from breast cancer patients. Hypothesizing that a higher CCC for collective dissemination-associated genes and for IBC-associated genes would be associated with retention of epithelial traits enabling collective dissemination and with worse disease progression in breast cancer patients, we evaluated the correlation of CCC with different phenotypic groups. The CCC of both the abovementioned gene sets, the collective dissemination-associated genes and the IBC-associated genes, was higher in (a) epithelial cell lines as compared to mesenchymal cell lines and (b) tumor samples from IBC patients as compared to samples from non-IBC breast cancer patients. A higher CCC of both gene sets was also correlated with a higher rate of metastatic relapse in breast cancer patients. In contrast, neither the levels of *CDH1* gene expression nor gene set enrichment analysis (GSEA) of the abovementioned gene sets could provide similar insights. These results suggest that retention of some epithelial traits in disseminating tumor cells as IBC progresses promotes successful breast cancer metastasis. The CCC provides additional information regarding the organizational complexity of gene expression in comparison to GSEA. We have shown that the CCC may be a useful metric for investigating the collective dissemination phenotype and a prognostic factor for IBC.

## Introduction

Metastasis is responsible for 90% of deaths from solid tumors (1). It involves the escape of cancer cells from the site of the primary tumor, their entry into the circulatory system, and finally, colonization of and proliferation at a distant organ. However, this process is highly inefficient. Only an estimated 0.2% of the disseminated tumor cells are able to form a lesion at distant organ sites (2, 3). A well-studied mechanism of metastasis is single cell dissemination where carcinoma cells acquire migratory and invasive traits *via* an epithelial-to-mesenchymal transition (EMT) (4). These cells can then utilize blood or lymph circulation to reach distant organ sites, where they reacquire epithelial traits of cell–cell adhesion and apico-basal polarity *via* a mesenchymal-to-epithelial transition (MET) to establish metastases (4).

Recent studies have highlighted that EMT is not a binary process. Rather, cells *en route* to a mesenchymal phenotype can acquire a stable hybrid epithelial–mesenchymal (hybrid E/M) phenotype (5, 6). These observations have called into question the indispensability of a complete EMT followed by MET in metastasis (7). Instead, collective migration of tumor cells *via* clusters of circulating tumor cells (CTCs) has been suggested as an alternate mechanism of metastasis (8). Clusters of tumor cells had been detected in the bloodstream of cancer patients even before the characterization of EMT as a driver of cancer metastasis (9, 10). These clusters of tumor cells can efficiently seed secondary tumors, exhibiting up to 50 times the metastatic potential of individually migrating tumor cells (11). Tumor cell clusters accounted for >90% of metastases in a mouse model of breast cancer (12). Abundance of CTC clusters in the bloodstream has been associated with significantly poor prognosis in breast cancer and in small cell lung cancer (SCLC) (11, 13).

Multiple factors are believed to be responsible for the heightened metastatic potential of these CTC clusters. These include effective response to mechanical signals and chemical gradients by cells in CTC clusters as compared to migrating single tumor cells (14, 15), better evasion of the host immune system (16), and potential cooperation among heterogeneous cell types in CTC clusters (17, 18). Studies have shown that collectively invading tumor cells from the primary lesion often co-express epithelial and mesenchymal markers (19–21). Thus, cells in CTC clusters tend to manifest a hybrid epithelial–mesenchymal (hybrid E/M) phenotype and to retain cell–cell adhesion characteristics (8).

Inflammatory breast cancer (IBC) is a highly aggressive breast cancer subtype that has been reported to predominantly metastasize *via* CTC clusters (22). Characterized by breast erythema, edema, and *peau d’orange* presenting with or without a noticeable tumoral mass (23, 24), IBC involves tumoral infiltrate in the dermal lymphatics and about 30% of IBC patients have distant metastases at the time of diagnosis as compared to only 5% of non-IBC type breast cancer patients (25). Though only 2–4% of breast cancer cases each year in the United States are of the IBC type, IBC patients account for 10% of the annual breast cancer-related mortalities. A hallmark of IBC is the presence of cohesive clusters of tumor cells in the local lymph nodes (26) and IBC patients have larger and a higher frequency of CTC clusters as compared to non-IBC breast cancer patients (27). Abundance of CTC clusters has been shown to be associated with poor progression-free survival in IBC patients (27). Despite their great propensity to metastasize, tumor cells in the primary lesion and in metastatic lesions of IBC maintain a high expression E-cadherin, a hallmark of epithelial cells (26). IBC thus presents an exciting model for the study of collective dissemination of tumor cells *via* CTC clusters and of the prognostic potential of these clusters of migrating tumor cells. The results presented here strengthen the argument for investigating IBC to elucidate the mechanisms underlying collective dissemination of tumor cells.

Here, we invoke concepts from theoretical models of evolution to investigate cluster-based dissemination of tumor cells and analogous IBC characteristics. Theoretical studies suggest that systems with a more hierarchical structure are more adaptable (28–30) due to their ability to efficiently span the space of possible states. Hierarchical systems are also more robust to perturbations because a hierarchical network architecture has a buffering effect that hinders the propagation of local perturbations to a majority of nodes (30, 31). Hierarchical organization, thus, emerges over time in physical systems that are evolving in a changing environment with a rugged fitness landscape exhibiting numerous peaks and valleys (29). Given that tumor cells involved in metastasis and invasion progress through many different microenvironments (32–34), one can expect the expression of genes associated with a metastatic phenotype to be more hierarchically organized in instances of successful macrometastases as compared to instances with no metastasis.

We quantified the hierarchical organization in the expression of two distinct sets of genes, one associated with collective dissemination of tumor cells and the other related to IBC, in cancer cell lines and in breast cancer patients. For this purpose, we used the cophenetic correlation coefficient (CCC) metric. The CCC for a set of genes takes into consideration the collective expression of all genes within the given set and the correlations between the expression levels of different genes. It captures the level of hierarchical organization in the collective expression of genes in the given set. A higher CCC indicates greater hierarchical organization in the expression of genes. The CCC was first used for comparing tree-like relationships represented by different dendrograms (35). It has been used previously to quantify the differences in expression of metastasis-associated genes in breast cancer patients with different clinical outcomes (36) and to quantify the differences in expression of genes predictive of clinical outcome in adult acute myeloid leukemia in patients belonging to different risk categories (37).

The goal of the present study was to determine whether the hierarchical organization in the expression of two sets of genes of interest is different in cell lines exhibiting different EMT-associated phenotypes and in tumor samples from breast cancer patients exhibiting features of IBC and non-IBC type disease. The first set of genes investigated here includes 87 genes reported to be associated with collective dissemination of tumor cells as CTC clusters: genes differentially expressed in cells forming CTC clusters as compared to individual CTCs (12). The second gene set includes 78 genes reported to be differentially expressed in tumor samples from IBC patients in comparison to tumor samples from non-IBC breast cancer patients (38). We observed that the CCC for both of these gene sets was higher in (a) epithelial cell lines as compared to mesenchymal cell lines and (b) tumor samples from IBC patients as compared to tumor samples from non-IBC breast cancer patients. A higher CCC further correlated with worse disease progression in breast cancer patients. In light of these observations, we propose that the metastatic aggressiveness of IBC potentially derives from the hierarchical organization in the expression of collective dissemination-associated genes in metastasizing tumor cells.

## Materials and Methods

### Genes Associated With Collective Dissemination of Tumor Cells

Using multicolor lineage tracking, Cheung et al. showed that polyclonal seeding by disseminated clusters of tumor cells is the dominant mechanism for metastasis in a mouse model of breast cancer (12). These clusters accounted for more than 90% of distant organ metastases in mice. Circulating tumor cell clusters were observed to be enriched in expression of the epithelial protein keratin 14 (K14), and 87 genes with enriched or depleted expression in K14^+^ primary tumor cells as compared to K14^−^ primary tumor cells were identified. Broadly, expression of adhesion complex-associated genes was enriched and that of MHC Class II genes was depleted in K14^+^ cells. We used this set of genes as a signature of the collective dissemination phenotype.

### Genes Associated With the IBC Phenotype

Van Laere et al. obtained tumor samples from patients with breast adenocarcinoma: 137 samples from IBC patients and 252 samples from patients with non-IBC type breast cancer (non-IBC) (38). IBC patients were selected in accordance with the consensus diagnostic criteria described by Dawood et al. (23). RNA from the tumor samples was hybridized onto Affymetrix GeneChips (HGU133-series) to obtain the corresponding mRNA expression profiles. Linear regression models were employed to identify a set of 78 IBC specific genes, which were differentially expressed in IBC tumor samples as compared to non-IBC tumor samples, independent of the molecular subtype of the tumor (38). We used this set of genes as a signature of the IBC phenotype in breast cancer patients. There were no genes common between this set of IBC-associated genes and the set of collective dissemination-associated genes described above. Both gene sets are available as Supplementary Material. The statistical methods used previously to obtain these gene sets are summarized in the Supplementary Material.

### Gene Expression Data From Different Cell Lines

We used two different datasets of gene expression in cell lines, each cell line classified as epithelial (E), mesenchymal (M), or hybrid epithelial–mesenchymal (hybrid E/M). The first dataset was from the study by Grosse-Wilde et al. (39), Gene Expression Omnibus (GEO) accession number GSE66527. A total of 24 clones established from HMLER cell lines [normal human mammary epithelial cells immortalized and transformed with hTERT and the oncogenes *SV40LT* and *RAS* (40)] were sorted into 13 *CD24*^+^/*CD44*^−^ E clones and 11 *CD24*^−^/*CD44*^+^ M clones. The E clones and the M clones displayed cobble-stone like morphology and dispersed, fibroblast morphology, respectively.

The second dataset included gene expression from the National Cancer Institute 60 anticancer drug screen (NCI60), which includes panels of cell lines representing nine distinct types of cancer: leukemia, colon, lung, central nervous system, renal, melanoma, ovarian, breast, and prostate (41). The 60 cell lines have been classified into epithelial (E) (*n* = 11), mesenchymal (M) (*n* = 36), and hybrid epithelial-mesenchymal (hybrid E/M) (*n* = 11) categories on the basis of protein levels of E-cadherin and Vimentin (42). The gene expression data for these cell lines obtained using the Affymetrix Human Genome U133A array platform were downloaded from the CellMiner database (43, 44).

### Gene Expression Data From Tumor Samples From IBC and Non-IBC Breast Cancer Patients

We used three different datasets of gene expression in tumor samples obtained from breast cancer patients. Each patient in the three datasets was diagnosed with either IBC or non-IBC type breast cancer (non-IBC). Iwamoto et al., GEO accession number GSE22597, collected tumor biopsies prospectively from 82 patients with locally advanced disease. A clinical diagnosis of IBC was made in 25 of these patients (45). Boersma et al., GEO accession number GSE5847, examined primary breast tumor samples from 50 patients, 15 of whom were diagnosed with IBC on the basis of the pathology and medical reports (46). Finally, Woodward et al., GEO accession number GSE45584, obtained tissue samples from core biopsies of breast tissue in 40 breast cancer patients, 20 IBC and 20 non-IBC (24).

In Iwamoto et al. and Woodward et al., IBC diagnosis was made in patients with clinical presentation of breast erythema and edema over more than one-third of the breast. In Boersma et al., nine IBC patients presented with erythema and edema, while six IBC patients exhibited pathology indicating dermal lymphatic invasion and tumor emboli.

The microarray platforms and the normalization techniques used previously to obtain the gene expression profiles for different cell lines and for tumor samples from cancer patients have been outlined in the Supplementary Material.

### Definition of Gene Network for Different Phenotypic Groups

For each phenotypic group, e.g., NCI60 cell lines labeled as epithelial (E) or patients in the Iwamoto et al. (45) dataset diagnosed with IBC, and gene set, e.g., the set of genes associated with IBC or the set of collective dissemination-associated genes, we defined a network with the genes as nodes and weighted edges between these nodes. The weight of the edge between gene *i* and gene *j* in the phenotypic group *G* was defined as

(1) $$l_{ij}^{G}=\left|\sum_{k\in G}\frac{\left(e_{i}^{k}-\mu_{i}^{G})(e_{j}^{k}-\mu_{j}^{G}\right)}{\sigma_{i}^{G}\sigma_{j}^{G}}\right|$$

Here, $e_{m}^{k}$ is the expression of gene *m* in the sample *k* (patient/cell line), ${µ}_{m}^{G}$ and $\sigma_{m}^{G}$ are the mean and SD of the expression of gene *m* in the phenotypic group *G*, respectively, and the summation is over all patients or cell lines belonging to the group *G*. This definition resulted in a fully connected network for each phenotypic group and gene set. Since Eq. 1 is symmetric in *i* and *j*, the networks obtained were undirected.

We constructed such networks for the epithelial and mesenchymal cell lines in the Grosse-Wilde et al. (39) dataset and for the epithelial, mesenchymal, and hybrid epithelial–mesenchymal cell lines in the NCI60 dataset. Such networks were also constructed for IBC and non-IBC breast cancer patients in the three breast cancer datasets, Iwamoto et al. (45), Woodward et al. (24), and Boersma et al. (46), using each of the two gene sets described above, genes associated with collective dissemination of tumor cells and genes associated with the IBC phenotype.

### Calculation of the CCC

To quantify the hierarchy in the expression of a set of genes in different groups of patients and cell lines, we used a metric called the CCC (35). The CCC is a measure of how well a hierarchical clustering of nodes in a network reproduces the distances between nodes in the original network. Intuitively, the CCC is a measure of how tree-like a network is. Since a tree topology is a prototypical hierarchical structure, a measure of the tree-like characteristic of a network allows us to aptly quantify the underlying hierarchy in the structure of the network.

For calculating the CCC of a given network, we defined the distance between nodes *i* and *j* as the Euclidean commute time distance (ECTD) between the two nodes, which is given by the square root of the mean first passage time taken by a random walker to travel from node *i* to node *j* and then back to node *i*. The ECTD between nodes *i* and *j* depends not only on the weight of the edge between nodes *i* and *j* but also on the number of different possible paths between the two nodes. The ECTD decreases as the number of possible paths between the two nodes increases, and increases if any path between the two nodes becomes longer (47). This makes the ECTD suitable for clustering tasks. As described before, the network obtained for each phenotypic group and gene set was undirected and fully connected. This ensures that the ECTD between any pair of nodes will be finite. For a network with *N* nodes, we generated a *N* × *N* matrix *D* such that *D_ij_* is the ECTD between nodes *i* and *j* (48). The matrix *D* was then used as an input to the average linkage hierarchical clustering algorithm (49), which generates a tree topology (*T*), i.e., a dendrogram, which best approximates the distances between the nodes of the network given by the matrix *D*. We then calculated the CCC as the correlation between the original pairwise distances and the corresponding distances in the tree topology:

(2) $$CCC=\frac{\sum_{i<j}(D_{ij}-d)(T_{ij}-t)}{\sqrt{\sum_{i<j}{\left(D_{ij}-d\right)}^{2}\sum_{i<j}{\left(T_{ij}-t\right)}^{2}}}$$

Here, $d=\langle D_{ij}\rangle$ is the mean of the original pairwise distances and $t=\langle T_{ij}\rangle$ is the mean of the pairwise distance in the tree topology. If the original network is hierarchical, the distances between nodes in the tree topology obtained *via* hierarchical clustering (*T*) will be highly correlated with the distances between nodes in the original network (*D*). Hence, the CCC will be high. However, if the original network lacks any hierarchical organization, this correlation will be weak, and the CCC will be low.

To test the sensitivity of the calculated CCC to the choice of ECTD as the network distance metric for hierarchical clustering, we alternatively defined the distance between node *i* and node *j* in the network as the resistance distance (50) between the two nodes. The resistance distance between any two nodes is given by the effective electrical resistance when a battery is connected across the two nodes. Like the ECTD, the resistance distance depends on all possible paths between nodes *i* and *j* and is, therefore, suited for clustering tasks. Using the resistance distance to create the matrix *D* where *D_ij_* is the resistance distance between the nodes *i* and *j*, we calculated the CCC as described above.

The CCC calculated for a network was normalized with respect to the CCC of random networks with the same set of nodes but re-distributed edge weights. For this, we generated 10 such random networks by shuffling entries in the matrix *D* and then calculated the average of the CCCs of these random networks (CCC_rand_). The normalized CCC was then defined as

(3) $${\text{CCC}}_{\text{norm}}\text{=}\frac{\text{CCC}-{\text{CCC}}_{\text{rand}}}{\text{1}-{\text{CCC}}_{\text{rand}}}$$

Finally, to obtain the error in the estimate of CCC_norm_, we used the bootstrap method (51). The method assumes that the distribution of gene expression in a patient or cell line group is the empirical distribution function of the observed expression in samples within the group. For a patient or cell line group with size *n*, we drew *n* samples from the group with replacement and calculated CCC_norm_ for the sampled group. This sampling process was repeated 100 times to obtain 100 CCC_norm_ values. The SE in the estimate of the CCC_norm_ for the group was then given as the sample SD of the 100 sampled CCC_norm_ values. All *p*-values were also calculated using the bootstrap method.

The MATLAB code used for calculating the CCC is available at https://github.com/st35/gene-network-CCC.

## Results

### Higher CCC for the Collective Dissemination-Associated Gene Network in Epithelial Cell Lines and in IBC Patients

**Figure 1:**
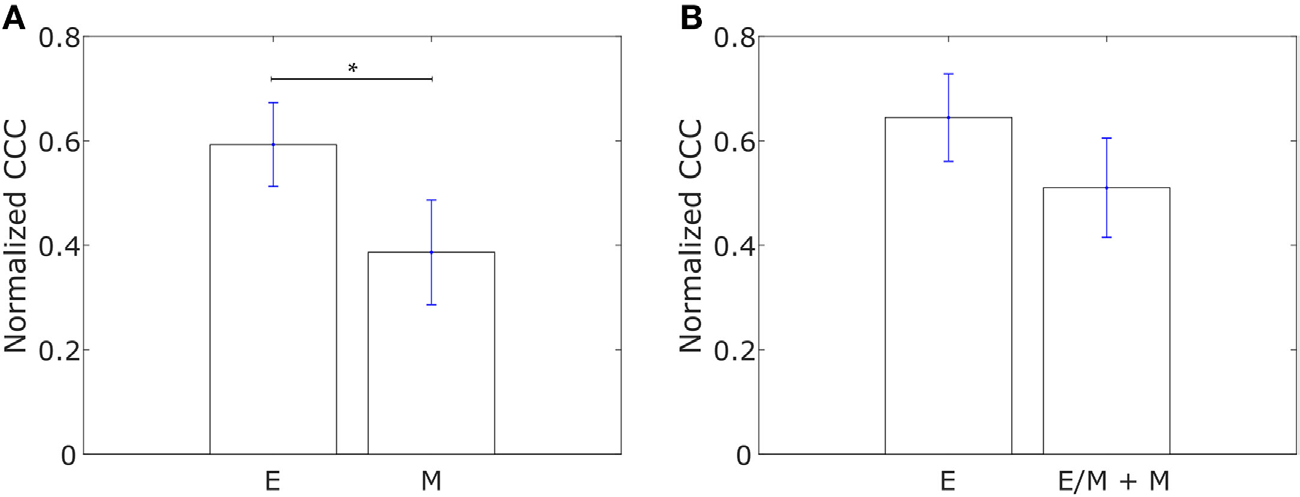
Normalized cophenetic correlation coefficient of the collective dissemination-associated gene network for **(A)** 13 epithelial (E) and 11 mesenchymal cell lines from the study by Grosse-Wilde et al. (39), and **(B)** 11 epithelial (E) and 47 hybrid epithelial-mesenchymal (E/M) + mesenchymal (M) cell lines from the NCI60 dataset (41, 42). Error bars indicate the SE in the estimate of CCC_norm_ calculated using the bootstrap method. *p-Value < 0.05.

We constructed networks with genes associated with collective dissemination of tumor cells (12), hereafter referred to as “collective dissemination-associated” genes, as nodes and the weight of the edge between nodes in a pair defined according to Eq. 1. Such networks were constructed for the E and M cell lines from the gene expression data from Grosse-Wilde et al. (39) and for the cell lines in the NCI60 anticancer drug screen (41) that have been categorized into E, M, and hybrid E/M classes (42). Representative networks for E and M cell lines from Grosse-Wilde et al. (39) are shown in Figures S1A,B in Supplementary Material. The normalized CCC for these networks was calculated using the method described above, and the results are shown in Figure 1. E cell lines exhibited a significantly higher CCC as compared to M cell lines (*p*-value = 0.01) for the collective dissemination-associated gene network in the dataset from Grosse-Wilde et al. (39), Figure 1A. In the NCI60 dataset, the normalized CCC of the collective dissemination-associated gene network was higher for E cell lines as compared to the pooled M and hybrid E/M cell lines, Figure 1B. The bootstrap distribution of normalized CCC values for E cell lines was distinct from the distribution for M cell lines in the dataset from Grosse-Wilde et al. (39) and from the distribution for pooled M and hybrid E/M cell lines in the NCI60 dataset (Kolmogorov–Smirnov test, *p*-value < 0.01). We further calculated the CCC using the resistance distance instead of the ECTD and observed a similar trend in CCC values in the two datasets, Figures S2A,C in Supplementary Material.

**Figure 2:**
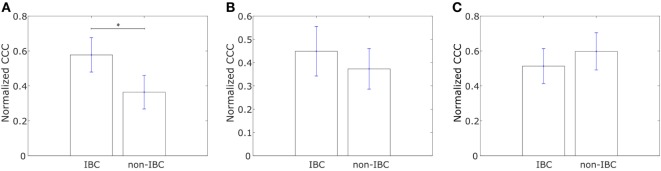
Normalized cophenetic correlation coefficient of the collective dissemination-associated gene network for tumor samples from inflammatory breast cancer (IBC) patients and non-IBC breast cancer patients for data from studies by **(A)** Iwamoto et al. (45) with 25 IBC and 57 non-IBC breast cancer patients, **(B)** Boersma et al. (46) with 13 IBC and 35 non-IBC breast cancer patients, and **(C)** Woodward et al. (24) with 20 IBC and 20 non-IBC breast cancer patients. Error bars indicate the SE in the estimate of CCC_norm_ calculated using the bootstrap method. *p-Value < 0.05.

We constructed similar networks for IBC and non-IBC patients using Affymetrix U133A profiles obtained by Iwamoto et al. (45). Representative networks for IBC and non-IBC breast cancer patients are shown in Figures S1C,D in Supplementary Material. Normalized CCC values for patients in the two groups are shown in Figure 2A. IBC patients exhibited a higher CCC for the network associated with collective dissemination of tumor cell clusters as compared to non-IBC breast cancer patients. The difference between the two groups in the dataset was significant (*p*-value < 0.02). Further, bootstrap distributions of the normalized CCC values for the two groups were statistically distinct with *p*-value < 0.01 for the Kolmogorov–Smirnov test. The same trend in CCC values was observed from calculation of CCC using the resistance distance, Figure S2E in Supplementary Material. However, we did not observe a significant trend for the breast cancer samples characterized by Boersma et al. (46) and for the samples characterized by Woodward et al. (24), Figures 2B,C.

### Higher CCC for the IBC-Associated Gene Network in Epithelial Cell Lines and in IBC Patients

**Figure 3:**
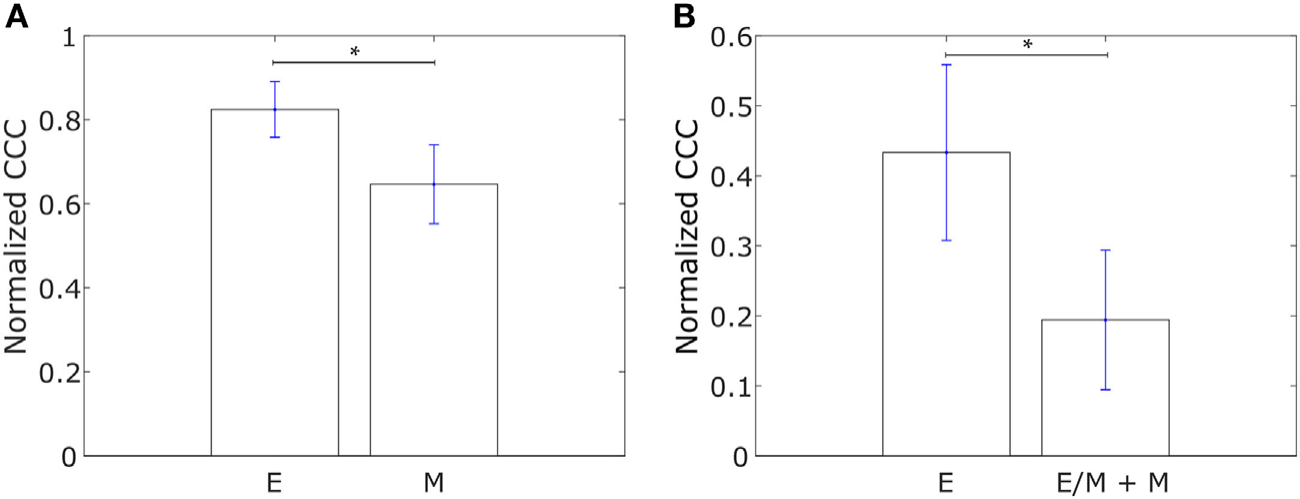
Normalized cophenetic correlation coefficient of the inflammatory breast cancer-associated gene network for **(A)** 13 epithelial (E) and 11 mesenchymal cell lines from the study by Grosse-Wilde et al. (39), and **(B)** 11 epithelial (E) and 47 hybrid epithelial-mesenchymal (E/M) + mesenchymal (M) cell lines from the NCI60 dataset (41, 42). Error bars indicate the SE in the estimate of CCC_norm_ calculated using the bootstrap method. *p-Value < 0.05.

We constructed networks with genes differentially expressed in tumor samples from IBC patients as compared to tumor samples from non-IBC breast cancer patients, hereafter referred to as “IBC-associated” genes, as nodes. The weight of the edge between nodes in a pair was defined using Eq. 1. Such networks were constructed for the E and M cell lines in the dataset from Gross-Wilde et al. (39) and for the E and pooled M + hybrid E/M cell lines in the NCI60 dataset. Normalized CCC values for these groups of cell lines calculated using the method described above are shown in Figure 3. E cell lines displayed a higher CCC for the IBC-associated gene network as compared to the other cell lines in both datasets [*p*-value = 0.03 for the cell lines in the study by Grosse-Wilde et al. (39) and *p*-value = 0.02 for the cell lines in the NCI60 dataset]. The bootstrap distributions of normalized CCC values for the two groups of cell lines were statistically distinct for both datasets (*p*-value < 0.01 for the Kolmogorov–Smirnov test in each case). A higher CCC for the epithelial cell lines was also observed on using the resistance distance to calculate the CCC, Figures S2B,D in Supplementary Material.

**Figure 4:**
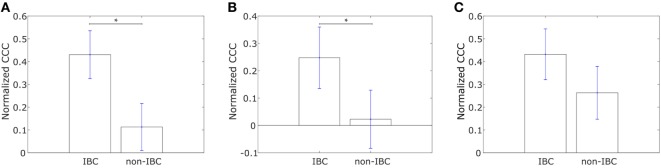
Normalized cophenetic correlation coefficient of the inflammatory breast cancer-associated gene network for tumor samples from IBC patients and non-IBC breast cancer patients for data from studies by **(A)** Iwamoto et al. (45) with 25 IBC and 57 non-IBC breast cancer patients, **(B)** Boersma et al. (46) with 13 IBC and 35 non-IBC breast cancer patients, and **(C)** Woodward et al. (24) with 20 IBC and 20 non-IBC breast cancer patients. Error bars indicate the SE in the estimate of CCC_norm_ calculated using the bootstrap method. *p-Value < 0.05.

Using Affymetrix U133A profiles from Iwamoto et al. (45), we constructed similar networks with IBC-associated genes as nodes for both IBC and non-IBC breast cancer patients. Normalized CCC values for the two breast cancer patient groups are shown in Figure 4A. The IBC group exhibited a significantly higher CCC for the IBC-associated gene network as compared to the non-IBC patient group (*p*-value = 0.01). Bootstrap distributions for the two groups were again statistically distinct (*p*-value < 0.01 for the Kolmogorov–Smirnov test). This trend in CCC values was also observed on using the resistance distance to calculate the CCC, Figure S2F in Supplementary Material. A similar trend in the CCC values for IBC and non-IBC patient groups was observed for breast cancer patients in the two other independent breast cancer datasets, Boersma et al. (46) (*p*-value = 0.02) and Woodward et al. (24) (*p*-value = 0.06), Figures 4B,C.

**Figure 5:**
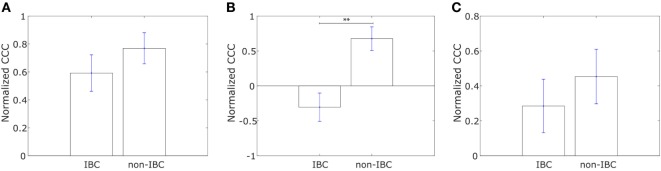
Normalized cophenetic correlation coefficient of canonical epithelial-to-mesenchymal transition driving transcription factors for tumor samples from inflammatory breast cancer (IBC) patients and non-IBC breast cancer patients for data from studies by **(A)** Iwamoto et al. (45) with 25 IBC and 57 non-IBC breast cancer patients, **(B)** Boersma et al. (46) with 13 IBC and 35 non-IBC breast cancer patients, and **(C)** Woodward et al. (24) with 20 IBC and 20 non-IBC breast cancer patients. Error bars indicate the SE in the estimate of CCC_norm_ calculated using the bootstrap method. **p-Value < 0.01.

Saunders and McClay had used a well-understood gene regulatory network in the sea urchin embryo to identify transcription factors that control cell changes during EMT by perturbing individual transcription factors (52). They further determined 30 human transcription factors homologous to those identified in sea urchins. We calculated the CCC of a network with these transcription factors, hereafter referred to as “canonical drivers of EMT,” as nodes for the IBC and non-IBC samples from each of the three breast cancer datasets, Iwamoto et al. (45), Boersma et al. (46), and Woodward et al. (24). The weight of the edge between any two transcription factors was defined using Eq. 1. We observed that the IBC patient group exhibited a lower CCC for the network composed of canonical EMT drivers as compared to the non-IBC patient group in data from each of the three studies, Figure 5.

### Higher CCC for the Two Networks Correlates With Early Metastasis Posttreatment

**Figure 6:**
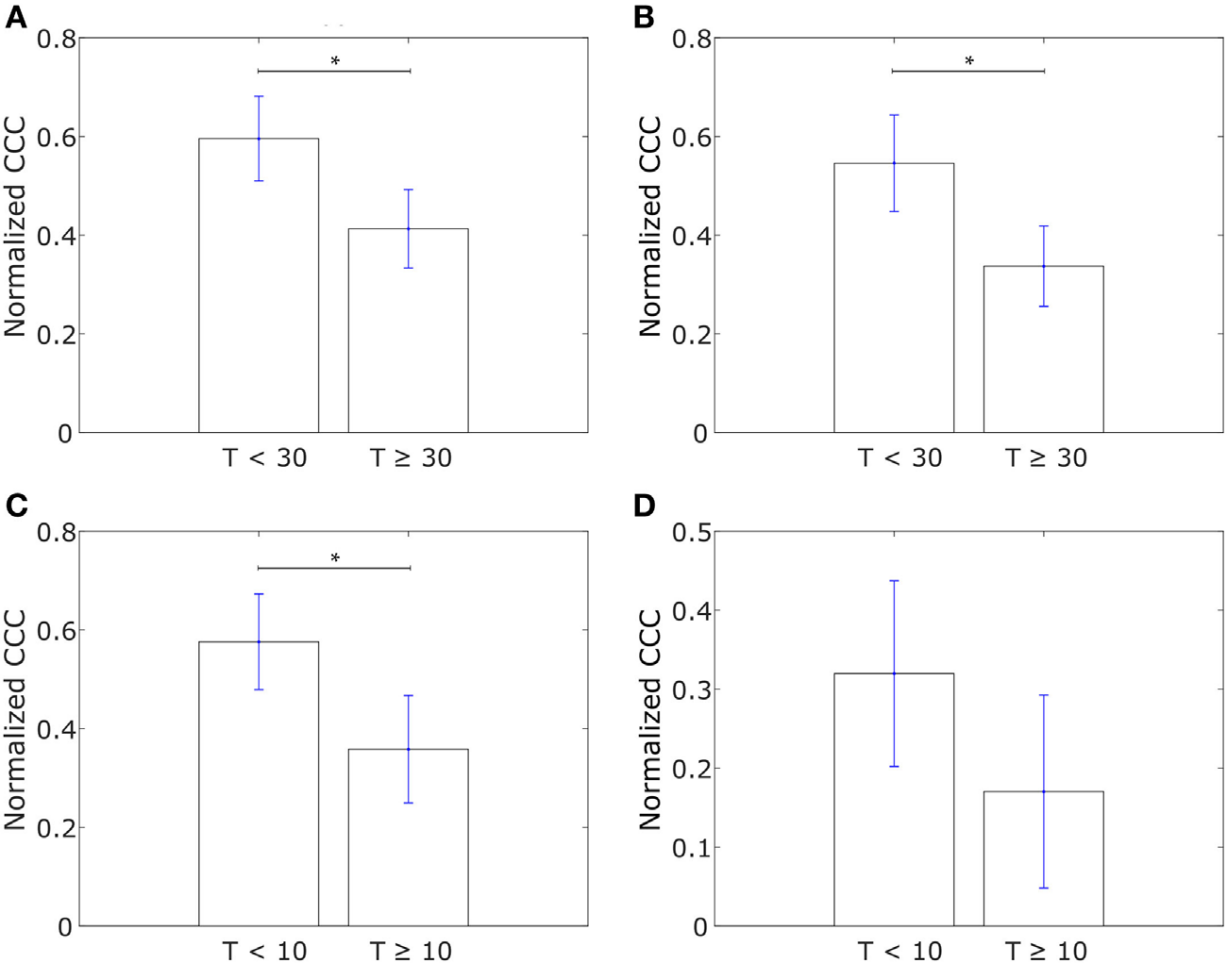
**(A)** Normalized cophenetic correlation coefficient of the collective dissemination-associated gene network for breast cancer patients with metastatic relapse within a 30-month period posttreatment (*T* < 30; *n* = 56) or between 30 and 60 months posttreatment (*T* ≥ 30; *n* = 51). Gene expression data from the study by Wang et al. (53). **(B)** Normalized CCC of the inflammatory breast cancer-associated gene network for the same groups of breast cancer patients as in **(A)**. **(C)** Normalized CCC of the collective dissemination-associated gene network for small cell lung cancer (SCLC) patients with less than 10 months of disease-free survival posttreatment (*T* < 10; *n* = 11) and SCLC patients with longer than 10 months of disease-free survival posttreatment but death during the follow-up period (*T* ≥ 10; *n* = 10). Gene expression data from the study by Rousseaux et al. (54). **(D)** Normalized CCC of the IBC-associated gene network for the same SCLC patient groups as in **(C)**. Error bars indicate the SE in the estimate of CCC_norm_ calculated using the bootstrap method. *p-Value < 0.05.

Having analyzed the differences in CCCs of collective dissemination-associated and IBC-associated gene sets in epithelial and mesenchymal cell lines and in tumor samples from IBC and non-IBC breast cancer patients, we next investigated if the CCC of these gene sets could provide insights into the metastatic propensity of tumors. We constructed networks with the two sets of genes, collective dissemination-associated and IBC-associated, as nodes for breast cancer patients who exhibited metastatic relapse within 5 years posttreatment (53). These patients were classified into two groups, those with metastatic relapse within 30 months posttreatment and those with metastasis between 30 and 60 months posttreatment, Figures 6A,B. Edge weights were defined, once again, using Eq. 1. For both collective dissemination-associated and IBC-associated gene sets, the CCC was significantly higher for the patient group with early metastatic relapse of breast cancer, i.e., relapse within 30 months of treatment, as compared to the patient group with relatively late relapse, i.e., metastatic relapse after 30 months posttreatment, Figures 6A,B. The *p*-values were 0.02 and 0.01 for the collective dissemination-associated gene network and the IBC-associated gene network, respectively. The same trend was observed upon considering only estrogen-receptor-positive patients, Figure S3 in Supplementary Material. There were too few samples from estrogen-receptor-negative patients for a similar analysis.

To investigate if the observation that a more hierarchical expression of collective dissemination-associated genes correlates with early relapse posttreatment can be generalized to other cancer types, we calculated the CCC of collective dissemination-associated and IBC-associated genes for SCLC patients. SCLC is a highly aggressive cancer subtype that is known to form tumor emboli and metastasize quickly, predominately *via* clusters of CTCs (55–57). SCLC patients with fewer than 10 months of disease-free survival posttreatment exhibited a higher CCC for both collective dissemination-associated and IBC-associated gene sets as compared to patients with greater than 10 months of disease-free survival posttreatment as computed from the data in the study by Rousseaux et al. (54), Figures 6C,D.

**Figure 7:**
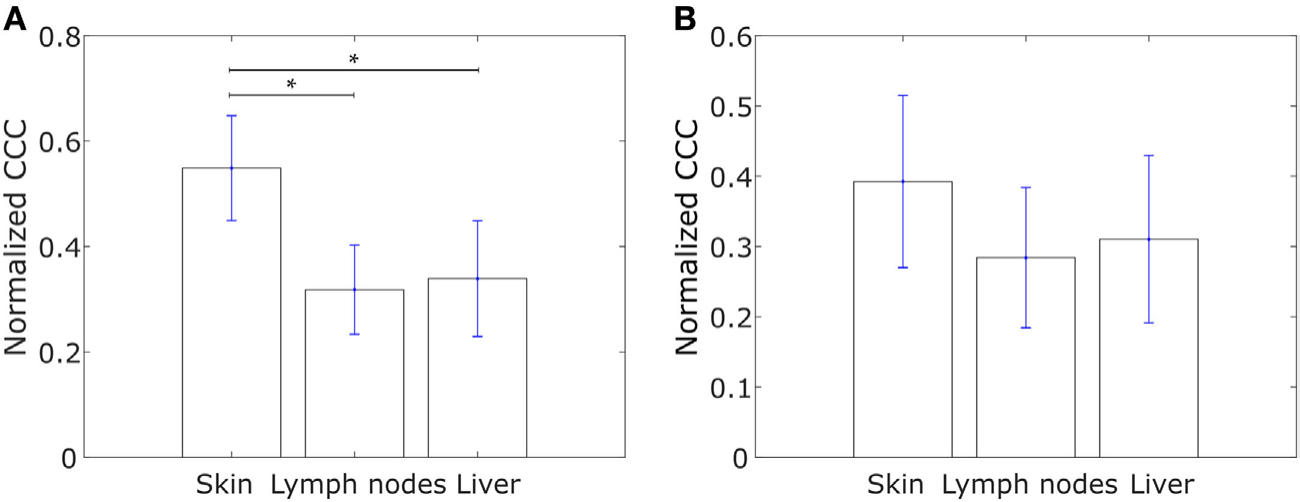
Normalized cophenetic correlation coefficient for tumor samples from breast cancer metastases to skin (*n* = 17), lymph nodes (*n* = 39), and liver (*n* = 16): **(A)** normalized CCC of the collective dissemination-associated gene network and **(B)** normalized CCC of the IBC-associated gene network. Gene expression data from the study by Kimbung et al. (59). Error bars indicate the SE in the estimate of CCC_norm_ calculated using the bootstrap method. *p-Value < 0.05.

The metastasis of cancer to different organs is characterized by organ-specific bottlenecks (58). While tumor cells from the site of the primary lesion can easily migrate to the local lymph nodes by moving passively with the lymph flow, migration to other organs such as skin or liver is much more challenging. Given the benefits afforded to migrating cancer cells by collective dissemination, cells with a more hierarchical expression of collective dissemination-associated genes are likely to be over-represented in cancer metastases to distant organs as compared to metastases to local lymph nodes. Using the gene expression data from the study by Kimbung et al. (59), we calculated the CCC of collective disseminated-associated genes in samples from breast cancer metastases to different organs and observed a higher CCC for metastases to skin as compared to metastases to lymph nodes and liver, Figure 7A. A similar trend was observed on calculating the CCC of the IBC-associated gene network, **Figure 7B**.

**Figure 8:**
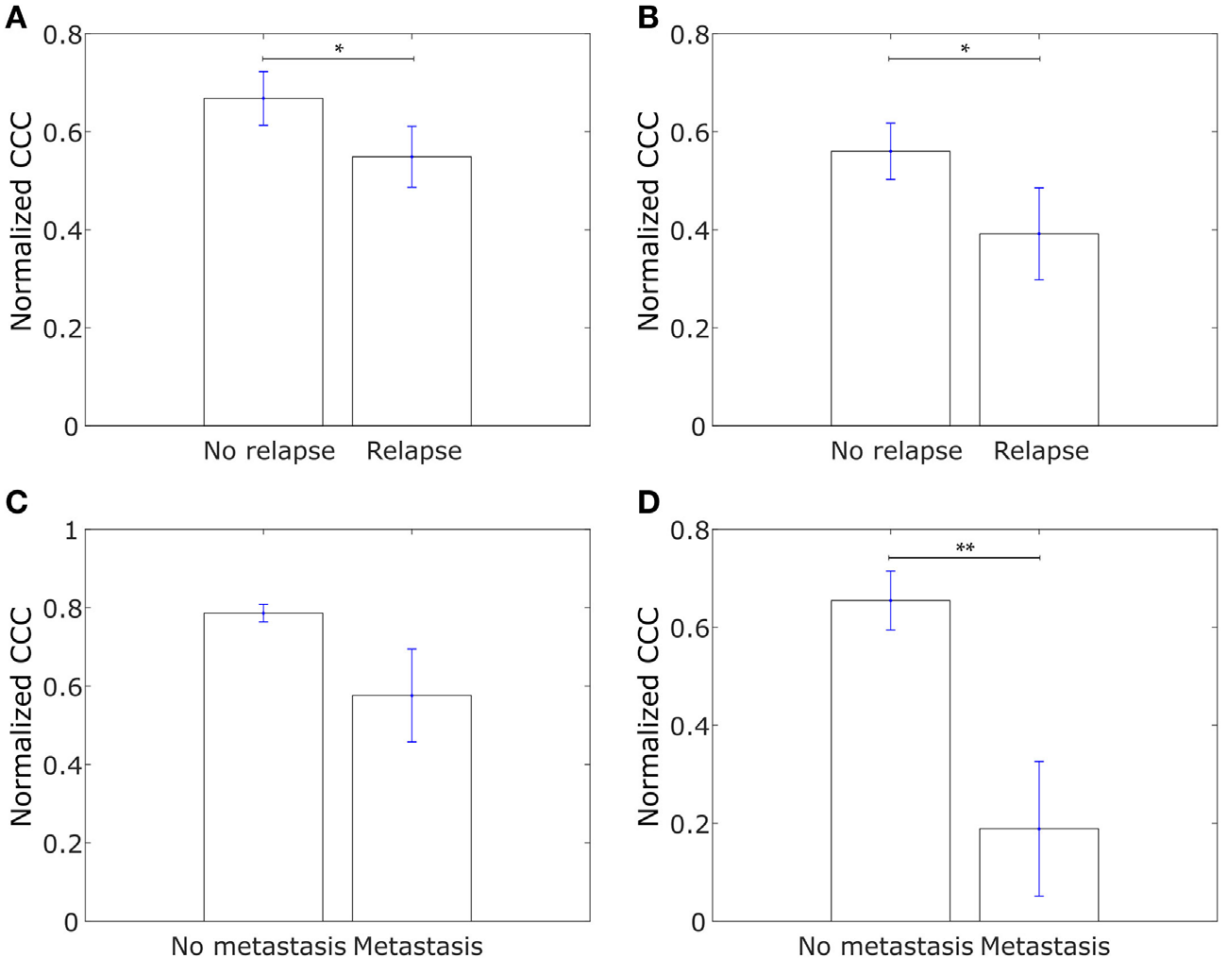
**(A)** Normalized cophenetic correlation coefficient of the collective dissemination-associated gene network for tumor samples from 107 breast cancer patients who did not exhibit breast cancer relapse during the follow-up period and for tumor samples from 179 patients who exhibited metastatic relapse during the follow-up period. Gene expression data from the study by Wang et al. (53). **(B)** Normalized CCC of the Inflammatory breast cancer-associated gene network for the same patient groups as in **(A)**. **(C)** Normalized CCC of the collective dissemination-associated gene network for tumor samples from 527 breast cancer patients with no metastasis during the follow-up period and for tumor samples from 13 patients with breast cancer metastasis during the follow-up period. Gene expression data from the cancer genome atlas (TCGA) (60). **(D)** Normalized CCC of the IBC-associated gene network for the same patient groups as in **(C)**. Error bars indicate the SE in the estimate of CCC_norm_ calculated using the bootstrap method. *p-value < 0.05 and **p-value < 0.01.

We further explored whether the CCCs for the collective dissemination-associated gene network and the IBC-associated gene network were different in breast cancer patients with metastatic relapse within 5 years posttreatment and those with no metastasis during this follow-up period as computed from the data in the study by Wang et al. (53). Intriguingly, we observed that the CCCs of both networks were significantly higher, *p*-value = 0.03 in each case, for patients with no metastasis during the 5-year follow-up period as compared to the patients with metastatic relapse during the follow-up, Figures 8A,B. A similar trend was observed for breast tumor samples from The Cancer Genome Atlas (TCGA) for patients who exhibited relapse during the follow-up period and those who did not (60), Figures 8C,D. Given that healthy breast cells are inherently epithelial, a higher CCC for the patient group with no metastatic relapse during the follow-up period may be a consequence of the tumor being at initial stages of progression toward a metastatic phenotype at the time of diagnosis and sample collection in these patient groups. However, upon grouping the breast cancer patients by their estrogen-receptor status, no consistent trend was observed between patients with no relapse during the follow-up period and patients with metastatic relapse during the follow-up period for both gene sets, Figure S4 in Supplementary Material. These results indicate that the collective dissemination pathway in breast cancer patients with differing receptor statuses warrants further study.

### The CCC Provides Additional Information Regarding the Underlying Complexity of Collective Gene Expression

**Figure 9:**
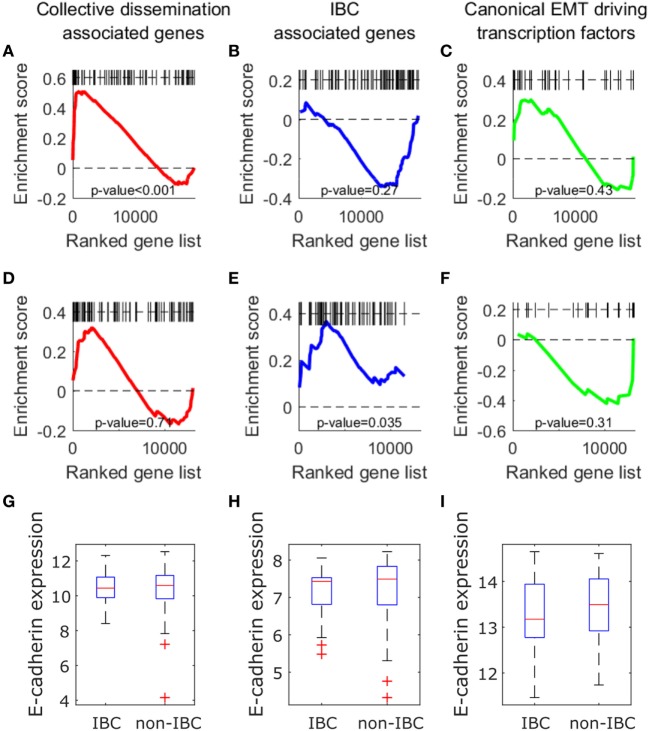
**(A–F)** Gene set enrichment analysis using collective dissemination-associated genes **(A,D)**, inflammatory breast cancer (IBC)-associated genes **(B–E)**, and canonical epithelial-to-mesenchymal transition driving transcription factors **(C–F)** on: **(A–C)** gene expression data for epithelial and mesenchymal cell lines from the study by Grosse-Wilde et al. (39) and on **(D–F)** gene expression data for tumor samples from IBC and non-IBC breast cancer patients from the study by Iwamoto et al. (45). In **(A–C)**, genes are ordered from left to right in decreasing order of correlation of expression with the epithelial phenotype. In **(D–F)**, genes are ordered from left to right in decreasing order of correlation of expression with the IBC phenotype. Black bars along the top of each plot indicate the positions of hits to the gene set along the ordered list of genes. Nominal *p*-values of enrichment are indicated at the bottom of each plot. **(G–I)** Mean expression of *CDH1* (E-cadherin) gene in tumor samples from IBC and non-IBC breast cancer patients in studies by **(G)** Iwamoto et al. (45), **(H)** Boersma et al. (46), and **(I)** Woodward et al. (24).

We next investigated if the insights described above can be obtained from an analysis of expression levels of collective dissemination-associated and IBC-associated genes. To determine how the CCCs of different gene networks correlate with the expression levels of these genes in different phenotypic groups, we carried out gene set enrichment analysis (GSEA) for different sets of genes on the epithelial and mesenchymal cell lines from the study by Grosse-Wilde et al. (39) and on the tumor samples from IBC patients and non-IBC breast cancer patients from the study by Iwamoto et al. (45). Using the GSEA software provided by the Broad Institute (61), we tested for enrichment in the expression of collective dissemination-associated genes, IBC-associated genes, and the canonical drivers of EMT in different phenotypic groups, i.e., epithelial versus mesenchymal cell lines in the data from Grosse-Wilde et al. (39) and IBC versus non-IBC patients in the data from Iwamoto et al. (45). The results are shown in Figures 9A–F. The expression of collective dissemination-associated genes was significantly enriched in epithelial cell lines as compared to mesenchymal cell lines (*p*-value < 0.001), Figure 9A, while IBC-associated genes and canonical EMT drivers did not show any such significant enrichment when compared across these two phenotypic groups. On the other hand, expression of IBC-associated genes was significantly enriched in tumor samples from IBC patients (*p*-value = 0.035), Figure 9E, while the collective dissemination-associated genes and canonical EMT drivers did not show significant enrichment on comparing IBC tumor samples with non-IBC breast tumor samples. We further divided the set of collective dissemination-associated genes into two groups, genes with enriched expression levels in K14+ cells and genes with depleted expression levels in K14+ cells. Neither of these two subsets exhibited significant enrichment when carrying out IBC tumor samples versus non-IBC breast tumor samples GSEA, Figure S5 in Supplementary Material.

Previous studies have suggested a strong association between expression of the E-cadherin protein in tumor cells and IBC (62, 63). To investigate if the level of *CDH1* (E-cadherin) gene expression in tumor samples from breast cancer patients is also associated with IBC, we compared the levels of *CDH1* gene expression in tumor samples from IBC and non-IBC patients. There was no significant difference in the expression levels of *CDH1* gene between the two patient groups in any of the three breast cancer patient datasets, Iwamoto et al. (45), Boersma et al. (46), and Woodward et al. (24), Figures 9G–I.

Finally, to test the specificity of the collective dissemination-associated and IBC-associated gene sets in characterizing IBC behavior, we generated 100 random gene sets. Each gene set consisted of 83 genes, average of the sizes of the collective dissemination-associated and IBC-associated gene sets. We calculated the normalized CCC of these gene sets in tumor samples from IBC and non-IBC breast cancer patients from the study by Iwamoto et al. (45). Only for 2 of the 100 randomly generated gene sets, the CCC was significantly higher for the IBC group as compared to the non-IBC group (*p*-value < 0.05), Figure S6 in Supplementary Material. This indicates that our hypothesis of a more hierarchically organized gene expression in IBC samples as compared to non-IBC breast cancer samples is specific to collective dissemination-associated and IBC-associated gene sets and is not applicable to randomly chosen sets of genes.

## Discussion

Cancer metastasis *via* migrating clusters of CTCs has emerged as a critical mechanism of seeding secondary tumors in recent studies (9–12). Although rare in comparison with individually disseminated cancer cells, CTC clusters can efficiently seed secondary tumors at distant organ sites (11, 12), and their presence in the bloodstream of cancer patients has been shown to be associated with poor disease prognosis, i.e., worse overall survival and worse disease-free survival (11). Understanding the molecular mechanisms underlying collective dissemination of tumor cells is, therefore, important for predicting metastasis, which remains the principal cause of cancer-associated mortalities. Determinants of single cell versus collective dissemination of tumor cells, however, remain elusive. Here, we have analyzed the topology of the network of genes implicated in the collective dissemination of tumor cells. We also investigated the topology of the network of genes reported to be differentially expressed in tumor samples from IBC patients as compared to tumor samples from non-IBC breast cancer patients. Taken together, our analysis suggests that maintenance of the epithelial phenotype in cancer cells disseminating from the primary tumor contributes toward metastasis *via* collective migration of tumor cells as CTC clusters.

Results suggest that the expression of genes differentially expressed in tumor cells migrating as clusters as compared to individually migrating tumor cells (12) exhibits a more hierarchical organization in epithelial cell lines as compared to mesenchymal cell lines among both, immortalized breast cancer cell lines (39) and the cancer cell lines in the NCI60 panel (41, 42). The importance of expression of such genes involved in cell migration, cell–extracellular matrix interaction, and cell–cell adhesion in the classification of NCI60 cell lines has been observed previously (64). Retention of some epithelial characteristics by cancer cells disseminating from the primary tumor has been reported to contribute toward collective invasion by tumor cells as CTC clusters (12, 65, 66). A more hierarchical organization in the expression of these genes may contribute toward a more robust epithelial phenotype in these cell lines (28–31). Higher hierarchical organization in the expression of these genes is also observed in tumor samples from IBC patients as compared to tumor samples from non-IBC breast cancer patients. This difference may contribute toward the strengthened presentation of epithelial characteristics such as cell–cell adhesion and juxtracrine signaling in tumor cells from IBC patients. The retention of these characteristics can foster the collective migration of tumor cells from the primary breast lesion (65). Further, our results reveal that hierarchical expression of collective dissemination-associated genes is of diagnostic relevance in IBC, thereby strengthening the case for IBC as a model system for the study of collective dissemination of tumor cells (22) and indicating the potential usefulness of mechanistic studies of tumor cell dissemination in determining the principles underlying IBC.

Next, we investigated the hierarchical organization in the expression of genes previously reported to be differentially expressed in tumor samples from IBC patients as compared to tumor samples from non-IBC breast cancer patients (38). The expression of these genes was more hierarchically organized in IBC samples as compared to non-IBC samples across multiple independent datasets. Further, epithelial cell lines exhibited a more hierarchical expression of these genes as compared to mesenchymal cell lines among immortalized breast cell lines (39) and among the cell lines in the NCI60 panel composed of nine different tumor types (41, 42). Thus, both collective dissemination-associated and IBC-associated genes exhibited a similar trend of higher CCC in immortalized breast cell lines or cancer cell lines as well as in tumor samples from IBC patients, adding to the existing evidence on collective dissemination *via* tumor emboli as the predominant mode of IBC metastasis and consequent aggressiveness. Intriguingly, the expression of canonical EMT-inducing transcription factors (52) was more hierarchically organized in non-IBC breast cancer samples as compared to IBC samples. Taken together, these results reinforce the notion that a complete EMT is not involved in IBC metastasis. Rather, it is the collective migration of tumor cells that are able to retain some epithelial characteristics that contributes toward the metastatic aggressiveness of IBC. The results presented here further strengthen the emerging notion that a complete EMT followed by MET is not necessarily as prevalent during cancer metastasis (7, 21) as posited earlier (67).

Both collective dissemination-associated and IBC-associated gene sets exhibited a higher CCC in breast cancer patients with faster posttreatment metastatic relapse as compared to patients with slower posttreatment relapse (53). A similar trend was observed in our calculations of the CCC for patients with SCLC (54), another metastatically aggressive cancer reported to metastasize *via* clusters of CTCs (55–57). These results indicate that a more hierarchical organization in the expression of genes involved in the collective dissemination of tumor cells may contribute toward a more aggressive behavior in metastatically aggressive tumors such as IBC and SCLC, which predominantly metastasize *via* clusters of CTCs. A mechanism-based investigation of the cross-talk between collective dissemination-associated and IBC-associated genes may, therefore, be a promising next step.

Further, samples from breast cancer metastases to lymph nodes and liver (59) exhibited a lower CCC as compared to breast cancer metastases to skin for collective dissemination-associated and IBC-associated gene sets (59). While metastasis of tumor cells to distant organs such as the skin is a complex, multi-step, and highly inefficient process, migration of tumor cells from the primary lesion to the local lymph nodes is likely to be a more facile process and can be brought about by the passive flow of the lymph. Metastasis to the liver is facilitated by the extravasation of migrating tumor cells into the liver *via* the fenestrated hepatic vascular epithelium (58). Correlation of the CCC for both gene sets, collective dissemination-associated and IBC-associated, with a higher rate of and propensity for metastasis to distant organs clearly speaks of the survival advantage afforded to migrating tumor cells by collective dissemination as clusters of CTCs. These advantages include enhanced ability to resist anoikis (cell death upon detachment from the substrate), evasion from immune system recognition, potential polyclonality, and enhanced ability to seed secondary tumors (68). In fact, CTC clusters can include non-tumor cells such as immune cells, platelets, and cancer-associated fibroblasts, thereby reproducing the primary tumor microenvironment conditions. Such an environment may contribute toward the survival of disseminating tumor cells in transit, promoting cancer metastasis (69).

A commonly used approach to determine if an *a priori* defined set of genes is associated with phenotypic differences between two groups is GSEA (70, 71). This method involves finding if the given set of genes is over-represented among genes that are differentially expressed in the two phenotypic groups. To determine if insights similar to those described above can be obtained *via* GSEA for the collective dissemination-associated gene set and for the IBC-associated gene set, we used the GSEA software provided by the Broad Institute (61) to calculate enrichment scores for the two gene sets in the data from Grosse-Wilde et al. (39), i.e., epithelial versus mesenchymal cell lines, and in the data from Iwamoto et al. (45), i.e., IBC versus non-IBC breast cancer patients. While we consistently obtained a higher CCC for collective dissemination-associated and IBC-associated gene sets in epithelial cell lines and in tumor samples from IBC patients, the expression of genes in these sets was not always enriched in epithelial versus mesenchymal cell lines or in IBC versus non-IBC patient samples. Together, these results indicate that the CCC need not correlate with GSEA. In fact, the CCC of a set of genes for two samples with a *k*-fold change in the expression of all genes in the set will be the same. The CCC can thus provide insights in addition to those that may be obtained from a direct analysis of gene expression data by using GSEA. The CCC of a gene network can be a robust metric of functional significance of a set of genes in different phenotypic groups, independent of the enrichment score calculated for the given gene set. It provides a prognostic measure based on the collective expression of genes in cells exhibiting different phenotypes beyond that provided by GSEA.

The classical view of cancer is that it involves de-differentiation of host cell pathways (36, 72). Since IBC is more metastatically aggressive as compared to non-IBC breast cancer, host cell pathways are likely to be more disrupted in tumor samples from IBC patients. This is indeed observed for breast tumor samples from the study by Iwamoto et al. (45). Of the 100 randomly generated gene sets, 41 exhibited a significantly higher CCC in the non-IBC breast cancer group as compared to the IBC group. This indicates that the host cell pathways are disrupted to a greater extent in IBC as compared to non-IBC breast cancer. However, structure in the pathways involving genes that promote cancer progression may be selected for as the disease advances. We previously showed that the expression of adult acute myeloid leukemia-associated genes is more hierarchically organized in samples from patients in whom the disease relapsed during the follow-up period as compared to patients that underwent complete remission upon treatment (37). Similarly, for breast cancer metastasis-associated genes, hierarchical organization was higher in patients who developed distant metastases during the follow-up period as compared to patients who did not (36). Here, we propose that due to the role of maintenance of an epithelial phenotype in collective dissemination of tumor cells and the subsequent metastatic efficiency of CTC clusters, a hierarchical organization in the expression of these genes may be selected for in metastatically aggressive cancers like IBC. A measure of hierarchical organization, here the CCC, can thus be a useful biomarker in cancer prognosis, particularly in the case of IBC.

## Conclusion

We have shown that a set of genes previously reported to be associated with the collective dissemination of tumor cells (12) is more hierarchically expressed in epithelial cell lines as compared to mesenchymal cell lines, thereby indicating a role for epithelial characteristics in the collective migration of tumor cells as clusters of CTCs. We further showed that IBC, an aggressive breast cancer subtype that metastasizes primarily *via* CTC clusters, exhibits a more hierarchical organization in the expression of these collective dissemination-associated genes as compared to non-IBC type breast cancer. Along similar lines, we showed that for genes differentially expressed in IBC as compared to non-IBC tumor samples, the expression is more hierarchical in tumor samples from IBC patients and in phenotypically epithelial cell lines, suggesting a a role for the retention of some epithelial traits in the metastatically aggressive nature of IBC. Taken together, our work indicates that at least some maintenance of the epithelial phenotype in disseminating tumor cells during disease progression plays a key role in successful metastasis of cancer to distant organs, and that IBC can be a suitable model system for studying mechanisms of collective migration of tumor cells as CTC clusters. Further, we have introduced the CCC as a quantitative metric for analyzing the collective migration of circulating tumor cell clusters, which can be useful in cancer prognosis, particularly in the case of IBC.

## Author Contributions

ST designed the study, carried out the analysis, and wrote the manuscript. MKJ designed the study, analyzed the results, and wrote the manuscript. WW and HL analyzed the results and edited the manuscript. MD supervised the study, analyzed the results, and edited the manuscript.

## Conflict of Interest Statement

The authors declare that the research was conducted in the absence of any commercial or financial relationships that could be construed as a potential conflict of interest.

## Supplementary Material

Figure S1 Representative collective dissemination-associated gene networks for **(A)** epithelial cell lines and **(B)** mesenchymal cell lines from the study by Grosse-Wilde et al. (39), and for **(C)** tumor samples from IBC patients and **(D)** tumor samples from non-IBC breast cancer patients from the study by Iwamoto et al. (45). The nodes are collective dissemination-associated genes and the weights of the edges between different nodes were defined using Eq. 1.

Figure S2 Normalized CCC of different gene sets calculated for different phenotypic groups using the resistance distance (2). Normalized CCC for 13 epithelial (E) and 11 mesenchymal cell lines from the study by Grosse-Wilde et al. (39), calculated using **(A)** collective dissemination-associated genes and **(B)** IBC-associated genes. Normalized CCC for 11 epithelial (E) and 47 epithelial–mesenchymal hybrid (E/M)+ mesenchymal (M) cell lines from the NCI60 dataset (41, 42), calculated using **(C)** collective dissemination-associated genes and **(D)** IBC-associated genes. Normalized CCC for tumor samples from the study by Iwamoto et al. (45) with 25 IBC and 57 non-IBC breast cancer patients, calculated using **(E)** collective dissemination-associated genes and **(F)** IBC-associated genes. Error bars indicate the SE in the estimate of CCC_norm_ calculated using the bootstrap method. **p*-Value < 0.05. The trend in CCC values observed here is same as the trend when calculating CCC using the Euclidean commute time distance, Figures 1, 2A, 3, and 4A.

Figure S3 Normalized CCC for estrogen-receptor-positive (ER+) breast cancer patients with metastatic relapse within a 30-month period posttreatment (*T* < 30; *n* = 36) or between 30 and 60 months posttreatment (*T* ≥ 30; *n* = 44): **(A)** normalized CCC of the collective dissemination-associated gene network and **(B)** normalized CCC of the IBC-associated gene network. Gene expression data from the study by Wang et al. (53). Error bars indicate the SE in the estimate of CCC_norm_ calculated using the bootstrap method. ***p*-value < 0.01. There were too few estrogen-receptor-negative (ER−) patients in the data set for similar analysis. The trend here is similar to the trend in Figure 6A.

Figure S4 Normalized CCC for breast cancer patients with different estrogen-receptor statuses. Patients with estrogen-receptor-positive status: **(A)** normalized CCC of the collective dissemination-associated gene network and **(B)** normalized CCC of the IBC-associated gene network. In this group, there were 129 patients with no relapse during the 5-year follow-up period and 80 patients with metastatic relapse posttreatment during the follow-up period. Patients with estrogen-receptor-negative status: **(C)** normalized CCC of the collective dissemination-associated gene network and **(D)** normalized CCC of the IBC-associated gene network. In this group, there were 50 patients with no relapse during the 5-year follow-up period and 27 patients with metastatic relapse posttreatment during the follow-up period. Gene expression data from the study by Wang et al. (53). Error bars indicate the SE in the estimate of CCC_norm_ calculated using the bootstrap method. **p*-Value < 0.05 and ***p*-Value < 0.01. In **(C)**, 10,000 bootstrap samples were drawn to calculate the normalized CCCs, obtain the error bars, and estimate the *p*-value.

Figure S5 Gene set enrichment analysis on gene expression data for tumor samples from IBC and non-IBC breast cancer patients from the study by Iwamoto et al. (45) using **(A)** genes upregulated in cells in circulating tumor cell clusters, and **(B)** genes downregulated in cells in circulating tumor cell clusters. Genes are ordered from left to right in decreasing order of correlation of expression with the IBC phenotype. Black bars along the top of each plot indicate the positions of hits to the gene set along the ordered list of genes. Nominal *p*-values of enrichment are indicated at the bottom of each plot.

Figure S6 Histogram of *p*-values calculated for the null hypothesis that the normalized CCC of a randomly generated gene set is higher in tumor samples from non-IBC breast cancer patients as compared to samples from IBC patients. Gene expression data from the study by Iwamoto et al. (45). Normalized CCC was calculated for 100 randomly generated gene sets consisting of 83 genes each. The red dotted line indicates *p*-value = 0.05 while the green dotted line indicates *p*-value = 0.95.
