# Supplementary figures and images for: Analysis of Hierarchical Organization in Gene Expression Networks Reveals Underlying Principles of Collective Tumor Cell Dissemination and Metastatic Aggressiveness of Inflammatory Breast Cancer
Source: Front Oncol. 2018 Jul 4;8:244. doi: 10.3389/fonc.2018.00244 (PMC6039554; doi:10.3389/fonc.2018.00244)

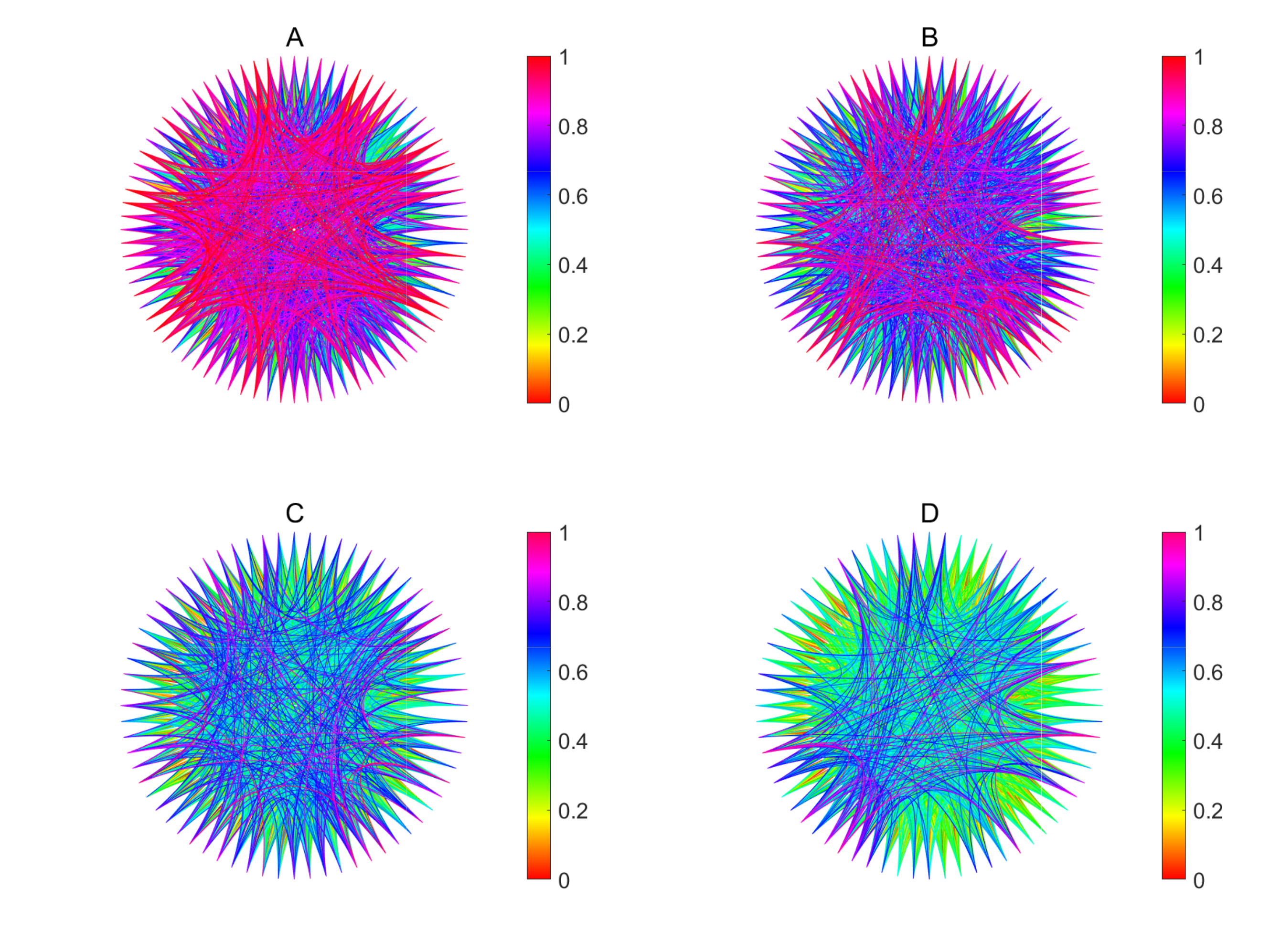

Supplement: Figure S1 — Representative collective dissemination-associated gene networks for (A) epithelial cell lines and (B) mesenchymal cell lines from the study by Grosse-Wilde et al. (39), and for (C) tumor samples from IBC patients and (D) tumor samples from non-IBC breast cancer patients from the study by Iwamoto et al. (45). The nodes are collective dissemination-associated genes and the weights of the edges between different nodes were defined using Eq. 1. [file image_1.tif]

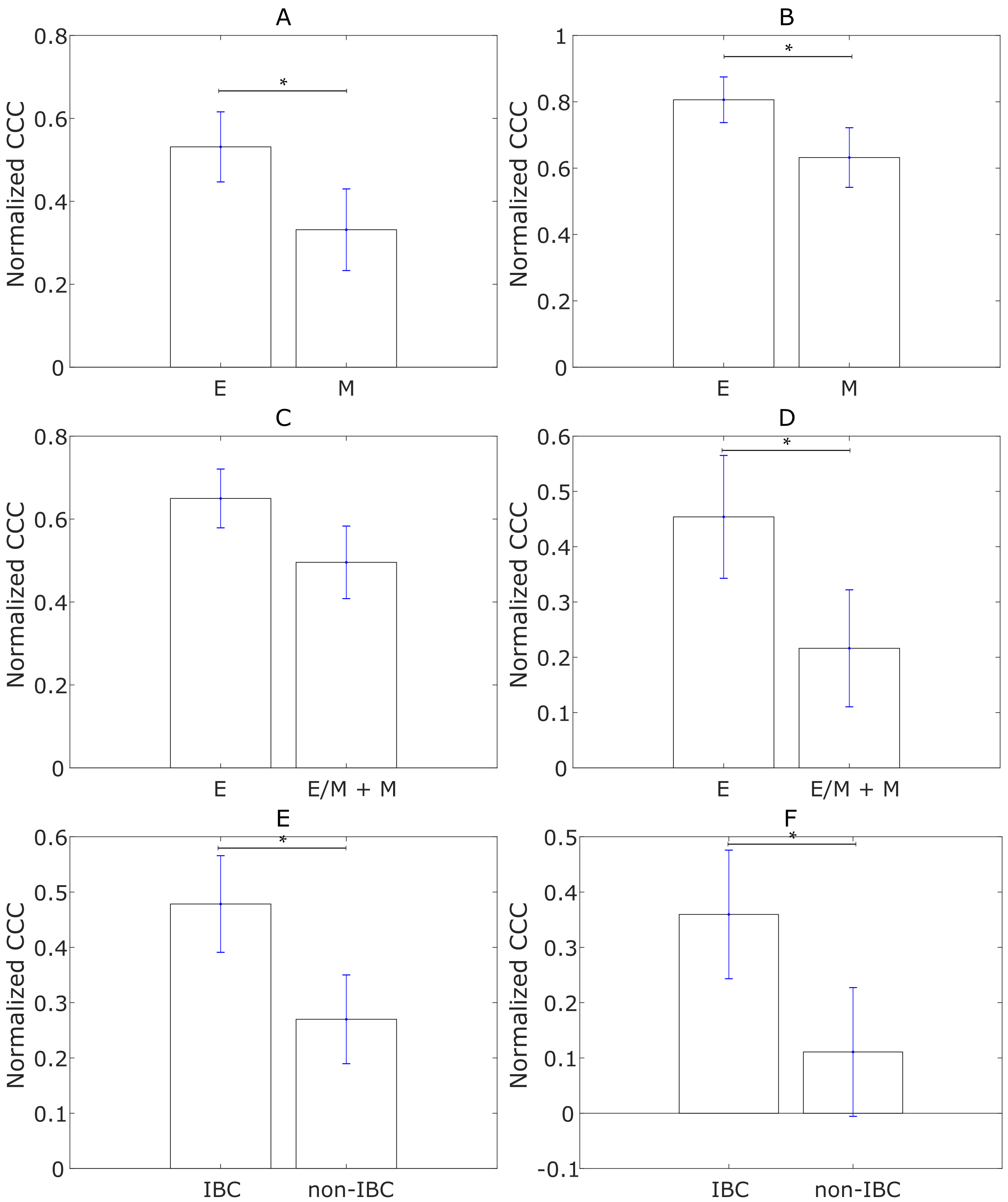

Supplement: Figure S2 — Normalized CCC of different gene sets calculated for different phenotypic groups using the resistance distance (2). Normalized CCC for 13 epithelial (E) and 11 mesenchymal cell lines from the study by Grosse-Wilde et al. (39), calculated using (A) collective dissemination-associated genes and (B) IBC-associated genes. Normalized CCC for 11 epithelial (E) and 47 epithelial–mesenchymal hybrid (E/M)+ mesenchymal (M) cell lines from the NCI60 dataset (41, 42), calculated using (C) collective dissemination-associated genes and (D) IBC-associated genes. Normalized CCC for tumor samples from the study by Iwamoto et al. (45) with 25 IBC and 57 non-IBC breast cancer patients, calculated using (E) collective dissemination-associated genes and (F) IBC-associated genes. Error bars indicate the SE in the estimate of CCCnorm calculated using the bootstrap method. *p-Value < 0.05. The trend in CCC values observed here is same as the trend when calculating CCC using the Euclidean commute time distance, Figures 1, 2A, 3, and 4A. [file image_2.tif]

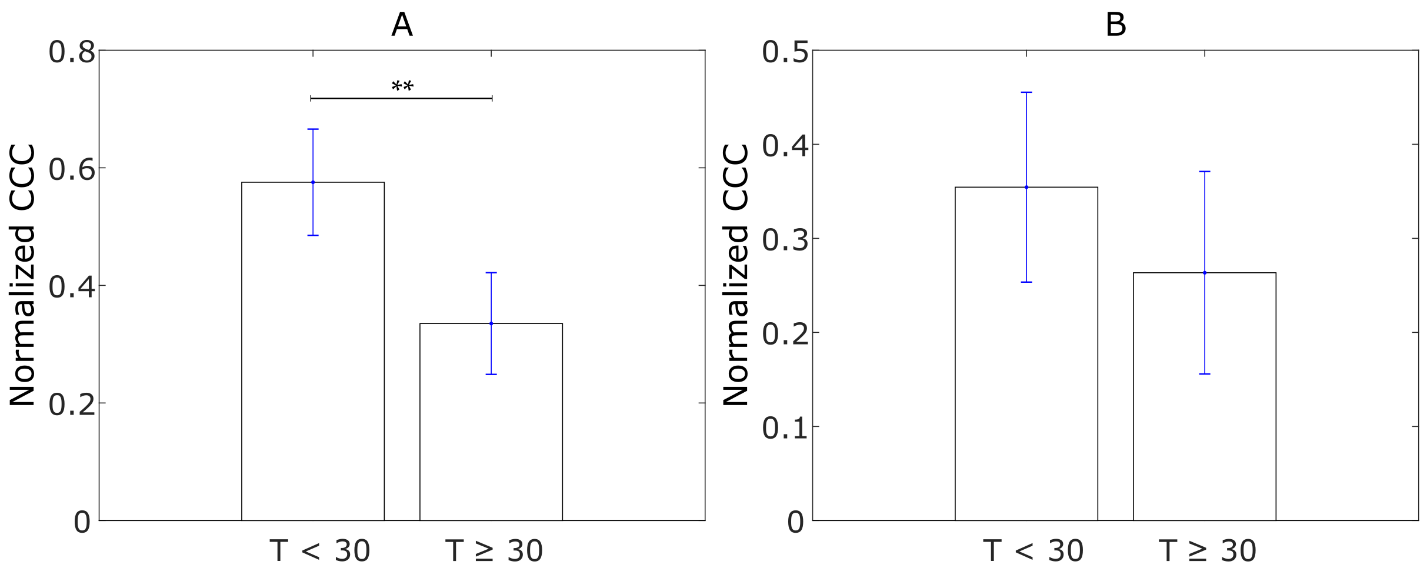

Supplement: Figure S3 — Normalized CCC for estrogen-receptor-positive (ER+) breast cancer patients with metastatic relapse within a 30-month period posttreatment (T < 30; n = 36) or between 30 and 60 months posttreatment (T ≥ 30; n = 44): (A) normalized CCC of the collective dissemination-associated gene network and (B) normalized CCC of the IBC-associated gene network. Gene expression data from the study by Wang et al. (53). Error bars indicate the SE in the estimate of CCCnorm calculated using the bootstrap method. **p-value < 0.01. There were too few estrogen-receptor-negative (ER−) patients in the data set for similar analysis. The trend here is similar to the trend in Figure 6A. [file image_3.tif]

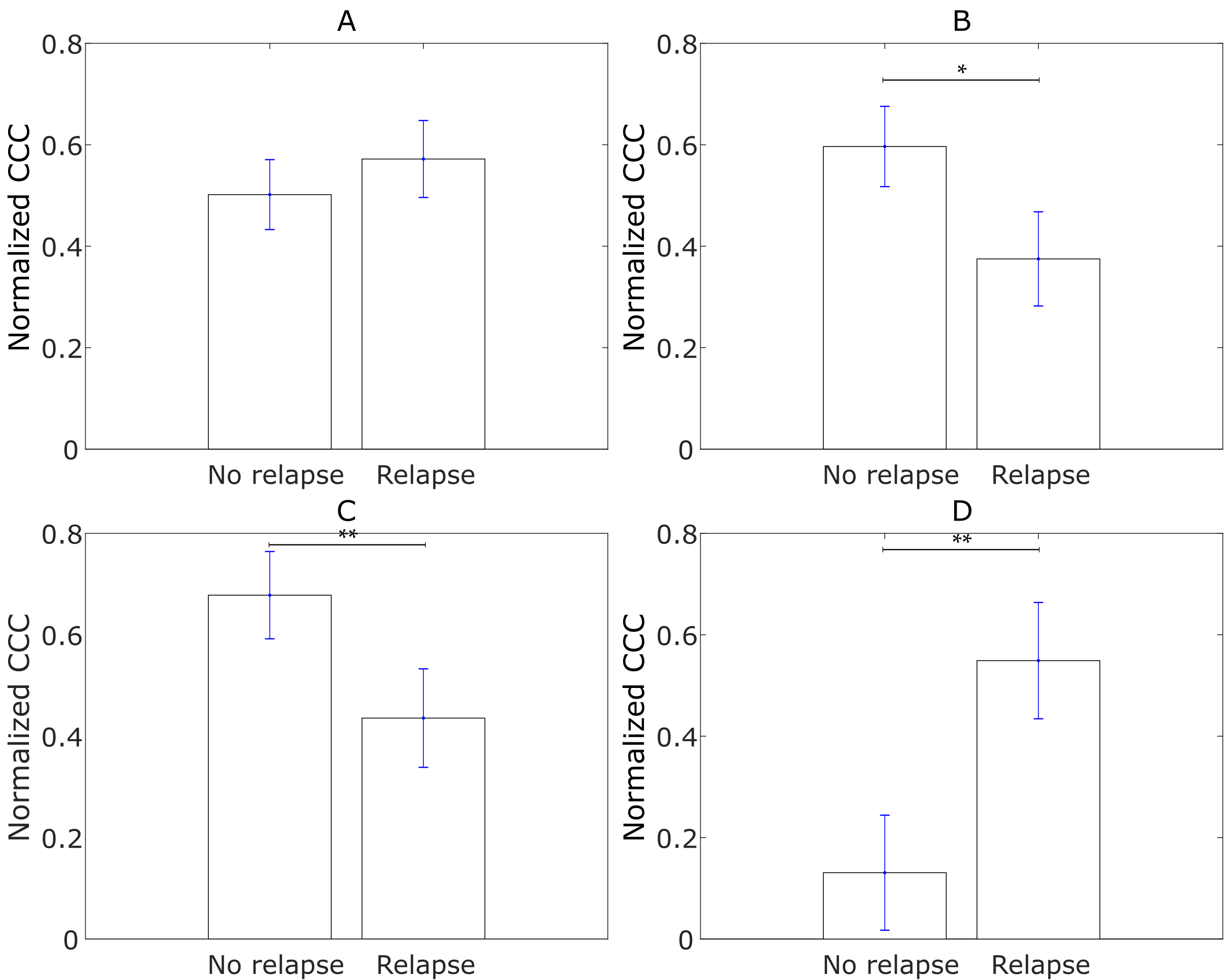

Supplement: Figure S4 — Normalized CCC for breast cancer patients with different estrogen-receptor statuses. Patients with estrogen-receptor-positive status: (A) normalized CCC of the collective dissemination-associated gene network and (B) normalized CCC of the IBC-associated gene network. In this group, there were 129 patients with no relapse during the 5-year follow-up period and 80 patients with metastatic relapse posttreatment during the follow-up period. Patients with estrogen-receptor-negative status: (C) normalized CCC of the collective dissemination-associated gene network and (D) normalized CCC of the IBC-associated gene network. In this group, there were 50 patients with no relapse during the 5-year follow-up period and 27 patients with metastatic relapse posttreatment during the follow-up period. Gene expression data from the study by Wang et al. (53). Error bars indicate the SE in the estimate of CCCnorm calculated using the bootstrap method. *p-Value < 0.05 and **p-Value < 0.01. In (C), 10,000 bootstrap samples were drawn to calculate the normalized CCCs, obtain the error bars, and estimate the p-value. [file image_4.tif]

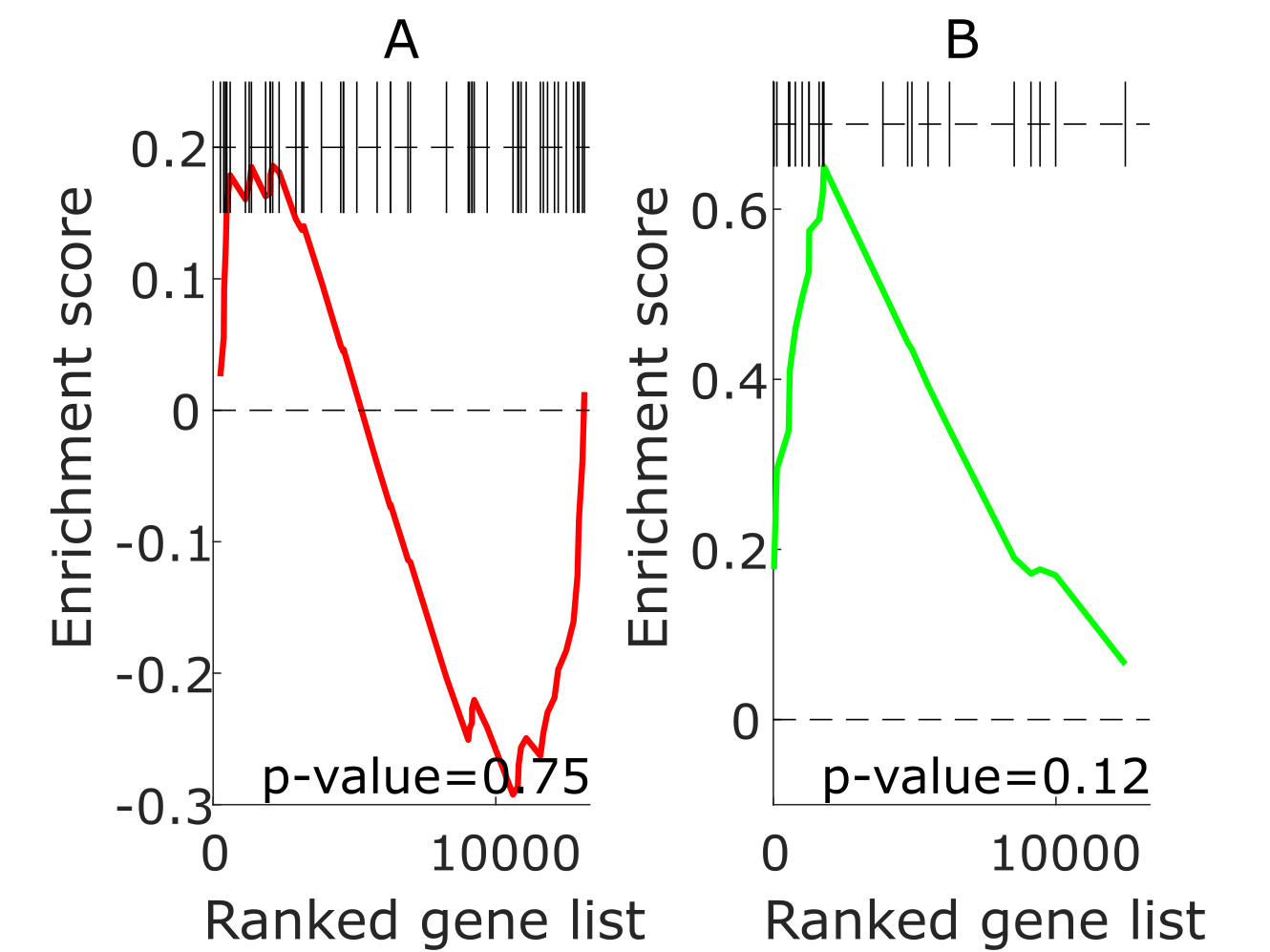

Supplement: Figure S5 — Gene set enrichment analysis on gene expression data for tumor samples from IBC and non-IBC breast cancer patients from the study by Iwamoto et al. (45) using (A) genes upregulated in cells in circulating tumor cell clusters, and (B) genes downregulated in cells in circulating tumor cell clusters. Genes are ordered from left to right in decreasing order of correlation of expression with the IBC phenotype. Black bars along the top of each plot indicate the positions of hits to the gene set along the ordered list of genes. Nominal p-values of enrichment are indicated at the bottom of each plot. [file image_5.tif]

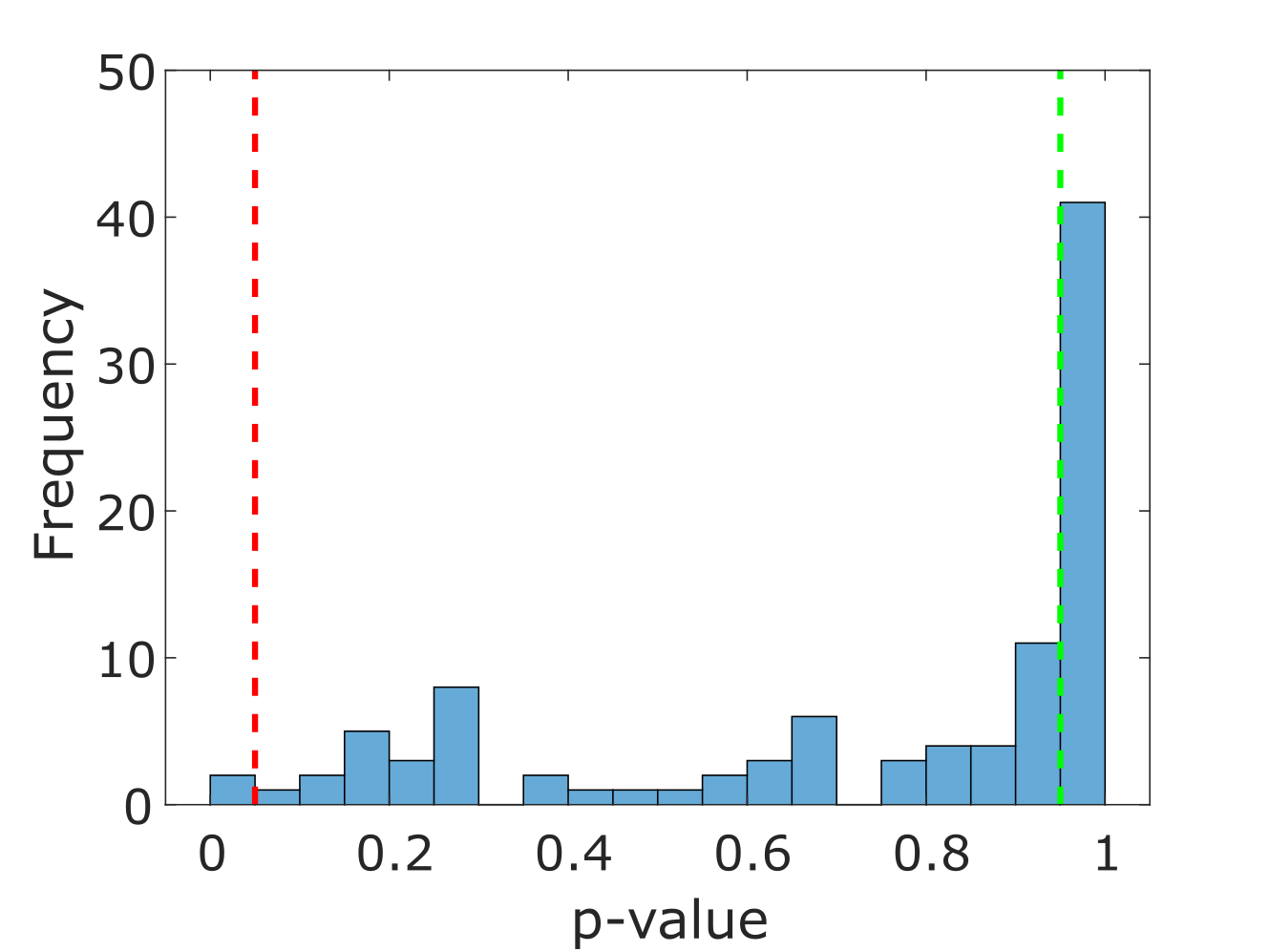

Supplement: Figure S6 — Histogram of p-values calculated for the null hypothesis that the normalized CCC of a randomly generated gene set is higher in tumor samples from non-IBC breast cancer patients as compared to samples from IBC patients. Gene expression data from the study by Iwamoto et al. (45). Normalized CCC was calculated for 100 randomly generated gene sets consisting of 83 genes each. The red dotted line indicates p-value = 0.05 while the green dotted line indicates p-value = 0.95. [file image_6.tif]
