# Supplementary material for: Analysis of Hierarchical Organization in Gene Expression Networks Reveals Underlying Principles of Collective Tumor Cell Dissemination and Metastatic Aggressiveness of Inflammatory Breast Cancer
Source: Front Oncol. 2018 Jul 4;8:244. doi: 10.3389/fonc.2018.00244 (PMC6039554; doi:10.3389/fonc.2018.00244)
Supplement: Supplementary file 7 [file data_sheet_1.docx]

**Supplementary information**

**Determination of the set of collective-dissemination associated genes**

Cheung *et al.* (1) observed that in a mouse model of breast cancer, locally disseminated tumor cell clusters, clusters of circulating tumor cells, and lung micrometastases were enriched for cells expressing the protein keratin-14 (K14) as compared to single disseminated cells. To compare the transcriptomes of K14^+^ and K14^-^ cells, Cheung *et al.* first isolated epithelial organoids from the primary MMTV-PyMT tumor (2,3) and used differential centrifugation to deplete immune and fibroblastic cells in order to focus on differences in gene expression between the two cancer cell subpopulations. The organoids were then processed into single cells and fluorescence-activated cell sorting (FACS) was used to isolate the K14^+^ and K14^-^ cells. RNA-seq reads were aligned to the mouse reference genome Genomic Reference Consortium build 38. A total of 4 such independent experiments were carried out. Number of reads mapped to each gene were determined using HTSeq v0.6.1p1 (4). Raw counts were then normalized and p-values for paired sample differential gene expression were calculated using EdgeR (4). Genes with less than 1 read per million in 4 of the 8 samples were excluded from further analysis. The procedure yielded a total of 12,190 mapped genes of which 11,968 had known annotations. DAVID Gene Set Analysis v6.7 with the functional annotation chart algorithm (5) was used for determining the Gene Ontology category enrichment, and p-values for GO category enrichment were calculated using a Fisher exact test. A total of 87 genes from GO categories functionally enriched or depleted in K14^+^ cell lines listed in table S1 of Cheung *et al.* (1) formed the set of collective dissemination-associated genes used in the present study.

**Determination of the set of IBC-associated genes**

Van Laere *et al.* (6) analyzed breast tumor samples from patients treated at 3 different institutions. After RNA-extraction from the 137 IBC and 252 non-IBC samples, hybridization onto Affymetrix GeneChips (HGU133-series) was carried out. Gene expression data were normalized by guanine cytosine robust multi-array analysis (7). Thereafter, in each of the 3 datasets (1 from each institution), probe sets with expression values above log_2_100 in at least 1% of the arrays were used for further analysis. A total of 9926 informative probes common to the 3 datasets were determined and used to merge the three datasets. Regression normalization was carried out using the Limma-package in BioConductor to remove technical, laboratory-specific variation in gene expression in the distinct datasets. To distinguish the variations in gene expression that discriminate between IBC and non-IBC type breast cancer from the variations discriminating between different breast cancer molecular subtypes such as luminal A, luminal B, basal-like, HER2-enriched, and normal-like, the dataset was divided into a training set with 250 samples (80 IBC and 166 non-IBC samples) and a validation set with 139 samples (53 IBC samples and 86 non-IBC samples). The training set was analyzed using linear regression models that incorporated the molecular subtype classification and the IBC/non-IBC classification to identify the probe sets associated with IBC-specific gene expression. Lists of probe sets were analyzed for expression differences between IBC and non-IBC samples in the validation set using a global test (8). Classifier models based on these lists of probe sets were constructed using the nearest shrunken centroid algorithm with 10-fold cross-validation to determine an appropriate $\delta$-value for minimizing the cross-validated training error rate. The performance of classifier models was also tested on more homogeneous sub-groups of tumor samples such as comparing IBC and non-IBC tumor samples with the luminal molecular subtype. Finally, a 79-gene IBC / non-IBC signature was identified from this supervised analysis. This set of genes formed the set of IBC-associated genes used in the present study.

**Gene expression profiles of epithelial and mesenchymal cell lines in the study by Grosse-Wilde *et al.***

Grosse-Wilde *et al.* (9) labeled the isolated total RNA using the one color Low Input Quick Amp Labeling Kit (Agilent). The labeled probes were run on Human 4x44K Microarrays (Agilent), and spot quantification was performed using Agilent’s Feature Extractor software. Data normalization was carried out in Genedata Analyst 7.0 (Genedata, Basel, Switzerland) using central tendency followed by relative normalization.

**Gene expression profiles of cell lines in the NCI60 panel**

Biotinylated cRNA was combined with a hybridization mix, applied to HG-U133A microarray, and hybridized overnight. Arrays were then washed and stained according to the manufacturer’s instructions and scanned on an Affymetrix GS2500 scanner. The MAS5 algorithm was used to generate signal intensities, and the expression values were normalized to a mean target level of 100 (10).

**Gene expression profiles of tumor samples in the study by Iwamoto *et al.***

Gene expression profiling was carried out using Affymetrix U133A gene chips. The data were normalized using the MAS5 algorithm, mean centered to 600, and log2 transformed (11).

**Gene expression profiles of tumor samples in the study by Boersma *et al.***

Boersma *et al.* (12) hybridized the labeled cRNA onto Affymetrix HG-U133A GeneChips. The data were normalized using the robust multichip analysis procedure ([www.bioconductor.org](http://www.bioconductor.org)).

**Gene expression profiles of tumor samples in the study by Woodward *et al.***

Woodward *et al.* (13) hybridized the labeled cRNA onto Whole Human genome 4x44K microarray (Agilent Technologies). Expression data were obtained using an Agilent Microarray Scanner, analyzed with Agilent’s Feature Extraction Software version 9.5.1, and normalized using global-normalization methods.

**Gene expression profiles of tumor samples in the study by Wang *et al.***

Wang *et al.* (14) isolated the total RNA, prepared biotinylated targets, and hybridized these onto Affymetrix U133a GeneChip. Gene expression data were calculated using Affymetrix GeneChip analysis software MAS 5.0. Chips with average intensity less than 40 and those with background signal greater than 100 were filtered out. All probes were scaled to a target intensity of 600.

**Gene expression profiles of tumor samples in the study by Rousseaux *et al.***

Rousseaux *et al.* (15) hybridized biotinylated cRNA onto GeneChip Human Genome U133 plus 2.0 array, scanned the GeneChips using the Affymetrix GeneArray scanner, and normalized the expression data using the Robust Multi-Array average (RMA) algorithm (Genespring software; Agilent).

**Gene expression profiles of tumor samples in the study by Kimbung *et al.***

Kimbung *et al.* (16) hybridized the isolated total RNA onto custom-made Affymetrix HuRSTA-2a520709 gene chips. Raw gene expression levels were normalized using the robust multichip average (RMA) algorithm. The data were log2 transformed and mean-centered across the entire dataset.

**Gene expression profiles of tumor samples in the TCGA dataset**

Agilent custom 244K whole genome microarrays were hybridized and the expression data were gene-median centered (17).

**References**

1. Cheung KJ, Padmanaban V, Silvestri V, Schipper K, Cohen JD, Fairchild AN, Gorin MA, Verdone JE, Pienta KJ, Bader JS, et al. Polyclonal breast cancer metastases arise from collective dissemination of keratin 14-expressing tumor cell clusters. *Proc Natl Acad Sci* (2016) **113**:201508541. doi:10.1073/pnas.1508541113

2. Guy CT, Cardiff RD, Muller WJ. Induction of mammary tumors by expression of polyomavirus middle T oncogene: a transgenic mouse model for metastatic disease. *Mol Cell Biol* (1992) **12**:954–961. doi:10.1128/MCB.12.3.954

3. Lin EY, Jones JG, Li P, Zhu L, Whitney KD, Muller WJ, Pollard JW. Progression to Malignancy in the Polyoma Middle T Oncoprotein Mouse Breast Cancer Model Provides a Reliable Model for Human Diseases. *Am J Pathol* (2003) **163**:2113–2126. doi:10.1016/S0002-9440(10)63568-7

4. Anders S, McCarthy DJ, Chen YS, Okoniewski M, Smyth GK, Huber W, Robinson MD. Count-based differential expression analysis of RNA sequencing data using R and Bioconductor. *Nat Protoc* (2013) **8**:1765–1786. doi:10.1038/nprot.2013.099

5. Huang DW, Sherman BT, Lempicki RA. Systematic and integrative analysis of large gene lists using DAVID bioinformatics resources. *Nat Protoc* (2009) **4**:44–57. doi:10.1038/nprot.2008.211

6. Van Laere SJ, Ueno NT, Finetti P, Vermeulen P, Lucci A, Robertson FM, Marsan M, Iwamoto T, Krishnamurthy S, Masuda H, et al. Uncovering the molecular secrets of inflammatory breast cancer biology: An integrated analysis of three distinct affymetrix gene expression datasets. *Clin Cancer Res* (2013) **19**:4685–4696. doi:10.1158/1078-0432.CCR-12-2549

7. Irizarry RA, Bravo HC, Irizarry RA, Irizarry R, Hobbs B, Collin F, Beazer-Barclay Y, Antonellis K, Scherf U, Speed T, et al. Exploration, normalization, and summaries of high density oligonucleotide array probe level data. *Biostatistics* (2003) **4**:249–264. doi:10.1093/biostatistics/4.2.249

8. Goeman JJ, Van de Geer S, De Kort F, van Houwellingen HC. A global test for groups fo genes: Testing association with a clinical outcome. *Bioinformatics* (2004) **20**:93–99. doi:10.1093/bioinformatics/btg382

9. Grosse-Wilde A, D’Hérouël AF, McIntosh E, Ertaylan G, Skupin A, Kuestner RE, Del Sol A, Walters KA, Huang S. Stemness of the hybrid epithelial/mesenchymal state in breast cancer and its association with poor survival. *PLoS One* (2015) **10**:e0126522. doi:10.1371/journal.pone.0126522

10. Shankavaram UT, Reinhold WC, Nishizuka S, Major S, Morita D, Chary KK, Reimers MA, Scherf U, Kahn A, Dolginow D, et al. Transcript and protein expression profiles of the NCI-60 cancer cell panel: an integromic microarray study. *Mol Cancer Ther* (2007) **6**:820–832. doi:10.1158/1535-7163.MCT-06-0650

11. Iwamoto T, Bianchini G, Qi Y, Cristofanilli M, Lucci A, Woodward WA, Reuben JM, Matsuoka J, Gong Y, Krishnamurthy S, et al. Different gene expressions are associated with the different molecular subtypes of inflammatory breast cancer. *Breast Cancer Res Treat* (2011) **125**:785–795. doi:10.1007/s10549-010-1280-6

12. Boersma BJ, Reimers M, Yi M, Ludwig JA, Luke BT, Stephens RM, Yfantis HG, Lee DH, Weinstein JN, Ambs S. A stromal gene signature associated with inflammatory breast cancer. *Int J Cancer* (2008) **122**:1324–1332. doi:10.1002/ijc.23237

13. Woodward WA, Krishnamurthy S, Yamauchi H, El-Zein R, Ogura D, Kitadai E, Niwa SI, Cristofanilli M, Vermeulen P, Dirix L, et al. Genomic and expression analysis of microdissected inflammatory breast cancer. *Breast Cancer Res Treat* (2013) **138**:761–772. doi:10.1007/s10549-013-2501-6

14. Wang Y, Klijn JG, Zhang Y, Sieuwerts AM, Look MP, Yang F, Talantov D, Timmermans M, Meijer-van Gelder ME, Yu J, et al. Gene-expression profiles to predict distant metastasis of lymph-node-negative primary breast cancer. *Lancet* (2005) **365**:671–679. doi:10.1016/S0140-6736(05)17947-1

15. Rousseaux S, Debernardi A, Jacquiau B, Vitte A-L, Vesin A, Nagy-Mignotte H, Moro-Sibilot D, Brichon P-Y, Lantuejoul S, Hainaut P, et al. Ectopic activation of germline and placental genes identifies aggressive metastasis-prone lung cancers. *Sci Transl Med* (2013) **5**:186ra66. doi:10.1126/scitranslmed.3005723

16. Kimbung S, Kovács A, Bendahl P-O, Malmström P, Fernö M, Hatschek T, Hedenfalk I. Claudin-2 is an independent negative prognostic factor in breast cancer and specifically predicts early liver recurrences. *Mol Oncol* (2014) **8**:119–128. doi:10.1016/j.molonc.2013.10.002

17. Koboldt DC, Fulton RS, McLellan MD, Schmidt H, Kalicki-Veizer J, McMichael JF, Fulton LL, Dooling DJ, Ding L, Mardis ER, et al. Comprehensive molecular portraits of human breast tumours. *Nature* (2012) **490**:61–70. doi:10.1038/nature11412
